# Autonomous Folding of Soft Matter From Living Polymers

**DOI:** 10.1002/adma.74348

**Published:** 2026-08-29

**Authors:** Jiahe Huang, Tuo Zhao, Shixi Zang, Deep Malu, Huajian Ji, Glaucio H. Paulino, Yuhang Hu

**Affiliations:** ^1^ School of Chemical and Biomolecular Engineering Georgia Institute of Technology Atlanta Georgia USA; ^2^ Department of Civil and Environmental Engineering Princeton University Princeton New Jersey USA; ^3^ Princeton Materials Institute (PMI) Princeton University Princeton New Jersey USA; ^4^ The George W. Woodruff School of Mechanical Engineering Georgia Institute of Technology Atlanta Georgia USA

**Keywords:** fabrication, materials science, polymer, soft matter, soft robotics

## Abstract

Origami provides a versatile framework for shape‐morphing, yet existing systems are limited to transitions between an initial state and a predetermined folding outcome, offering little tunability after fabrication. Here we introduce a dynamic “living” polymer that continuously remodels its main‐chain network through a growth process, enabling post‐fabrication control over size, mechanical properties, and geometry. Incorporated as active crease layers, these polymers drive autonomous folding when supplied with “nutrient solutions” containing monomers, crosslinkers and catalysts. The associated dihedral angles are determined by growth kinetics and crease composition. Iterative regrowth reprograms folding pathways and increases mechanical stiffness up to 50 times. We demonstrate rigid (Miura) and non‐rigid patterns (square‐twist) that can be repeatedly reconfigured. Through this work, we establish a foundation for adaptive structures with unlimited re‐programmability, opening avenues for applications in biomedical wearable devices and multifunctional deployable systems.

## Graphical abstract

Origami structures are endowed with “living” polymer creases that grow, remodel, and stiffen in response to nutrient solutions. This post‐fabrication growth enables autonomous folding, iterative regrowth, and mechanical tuning involving origami assemblies, such as cranes, Miura‐ori tessellations, rigid and non‐rigid square‐twist patterns, establishing a route toward adaptive and reconfigurable soft matter with evolving geometry and functionality.

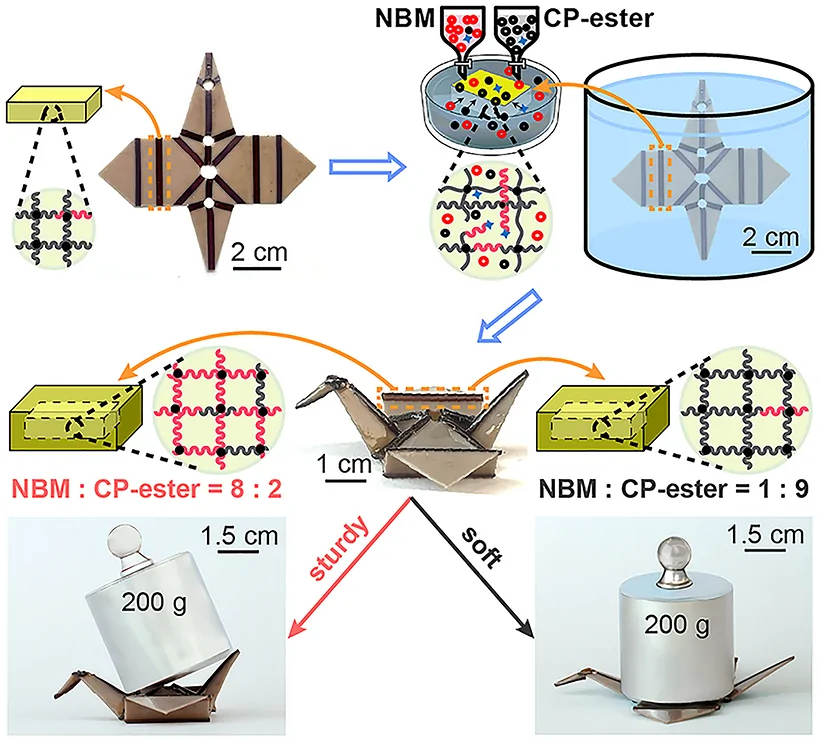

## 1 Introduction

Origami—once solely the art of paper folding—has become a powerful platform for programmable shape‐morphing across scales. By prescribing crease geometry and mountain–valley assignments, flat 2‐D sheets can transform into complex 3‐D architectures that are deployable, scalable, and reconfigurable [1]. These capabilities have already inspired technologies ranging from deployable solar arrays [2, 3], space‐borne optical shades [4], biomedical devices [5, 6], tissue engineering [7], soft robotics [8, 9], adaptive buildings [10], and artificial muscle [11]. Yet most origami structures still rely on manual folding, bulky actuators [12, 13], or pneumatic systems [14, 15], which significantly restrict miniaturization and remote operation.

Stimuli‐responsive polymers offer a more elegant route to self‐folding. Hydrogels can contract and swell in response to temperature shifts [16, 17], chemical cues [18, 19], and light irradiation [20, 21]; shape‐memory polymers change shape when heated [22, 23, 24]; liquid‐crystal elastomers reversibly deform with temperature [25, 26, 27, 28, 29]; and magnetically patterned elastomers fold within milliseconds under a magnetic field [30, 31, 32]. These materials have enabled diverse self‐folding geometries, reconfigurable origami, remotely actuated soft robots, and various origami machines. Yet despite their promise, current active polymers are confined to prefabricated main‐chain networks that restrict deformation to a few predefined states. Furthermore, traditional stimuli‐responsive materials often require a continuous supply of external energy, such as heat or light, to maintain intermediate configurations upon deformation. As a result, they provide little capacity for post‐fabrication tuning of size, mechanical properties, or folding pathways. These constraints fundamentally limit the reconfigurability and re‐programmability of origami structures built from them.

Here we introduce a dynamic “living” polymer and integrate its post‐fabrication reconfigurability directly into origami design. Unlike conventional active polymers, which only reorganize a fixed polymer network, this material can change its main‐chain architecture after fabrication, enabling growth, remodeling, and substantial changes in mechanical properties that resemble key behaviors of living systems. These capabilities enable continuous and extensive reprogramming of the size, mechanics, and folding pathways of origami structures. The resulting reprogrammed folding states can be individually maintained to support long‐term functionality without the continuous supply of external energy required by many stimuli‐responsive polymers. Synthesized via ring‐opening metathesis polymerization (ROMP) of cyclopentene and norbornene derivatives, the polymer chains retain double bonds suitable for further cross‐metathesis, allowing continuous “growth” when immersed in a “nutrient” solution containing monomers, crosslinkers, and catalysts. The concurrent “nutrient” uptake, polymerization, and main‐chain remodeling cooperatively enlarge and reconfigure the network, allowing its properties to be tuned simply by adjusting “nutrient composition”. When integrated as crease layers in bilayer origami, these “living” polymers drive self‐folding transitions from 2D to 3D with folding angles and stiffness programmable through growth chemistry. Crucially, this process is *iterative*: repeated nutrient treatments enable continuous reshaping and mechanical reprogramming, a capability absent in prior origami systems. By uniting the chemomechanics of “living” polymers with origami design principles, our work establishes a fabrication platform for adaptive materials with essentially unlimited tunability—an advance that opens new opportunities in soft robotics, biomedical devices, and deployable systems.

## 2 Results and Discussion

### 2.1 The Growth of “Living” Polymers and Growth‐Enabled Origami Self‐Folding

The new dynamic “living” polymer leverages open‐system and nonequilibrium conditions, moving beyond conventional equilibrium‐based closed‐system strategies. As illustrated in Figure 1a, we begin with a polymer whose main chain contains dynamic bonds that can be selectively activated to permit post‐fabrication modification. These polymers were synthesized by copolymerizing methyl cyclopent‐3‐ene‐1‐carboxylate (CP‐ester) and 5‐norbornene‐2‐methanol (NBM), together with cyclopentene‐based crosslinkers. CP‐ester and NBM were carefully chosen for a few reasons: both undergo ROMP chemistry enabling their random incorporation into a single network; the resulting polymer retains unsaturated double bonds allowing for postfabrication insertion of monomers and chain exchange via cross‐metathesis [33], both monomers are liquid at room temperature enabling direct diffusion and swelling into the network; they are mutually soluble; and they provide vastly different mechanical properties allowing precise tuning of material behaviors through adjustment of the NBM:CP‐ester ratio. When immersed in a “nutrient” solution containing CP‐ester, NBM, crosslinker, and Grubbs’ second‐generation catalyst (G2), the network absorbs these species and incorporates them into the original polymer backbones, thereby enabling growth. As the “nutrients” in the network are consumed, concentration gradients continuously drive further transport of “nutrients” into the network, sustaining the growth process. This process couples automatic and concurrent “nutrient” uptake, polymerization, chain exchange via cross‐metathesis, and remodeling of main‐chain elasticity. After a defined growth period, the polymer is washed and dried to yield a stable “grown polymer”. Beyond dimensional increase, the mechanical properties of the “grown polymer” can also be precisely tuned by adjusting the chemical composition of the nutrient solution, particularly the NBM:CP‐ester ratio. For example, two identical precursor polymers (made of 10% NBM, 88% CP‐ester, 2% crosslinker) grown in solutions of NBM:CP‐ester = 8:2 and 1:9 develop distinct mechanical properties (Figure 1a). The “8:2” solution contains 80% NBM, 18% CP‐ester, 2% crosslinker, and 8 mg/mL G2, whereas the “1:9” solution contains 10% NBM, 88% CP‐ester, 2% crosslinker, and 8 mg/mL G2. After growing to a similar mass (600% of initial mass), films grown in the 8:2 solution for 400 min exhibit a Young's modulus of 48 MPa (from the initial linear regime) and a breaking strain of 600%, much tougher and stiffer than the polymer grown in 1:9 solution for 180 min, which shows a modulus of 1.1 MPa and a breaking strain of 400%. The difference likely arises from the bulky appendant ring of NBM, which restricts chain mobility after polymerization and leads to a more rigid backbone compared to CP‐ester. In addition, NBM‐ containing chains can form hydrogen bonds, further enhancing the material's stiffness and toughness. Additional experimental details are provided in the “Growth of ‘living’ polymers” section of the Experimental Section.

**FIGURE 1:**
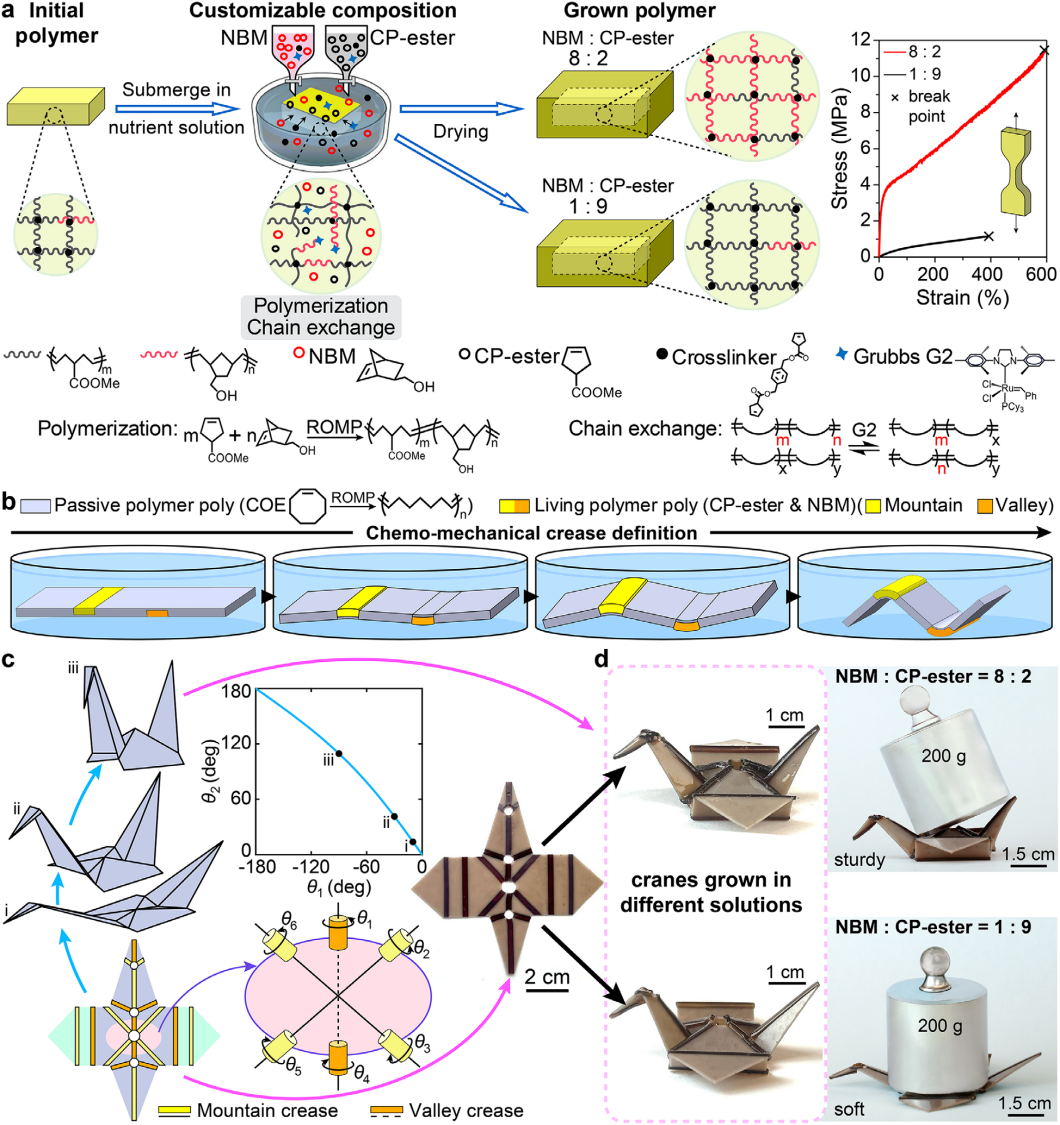
Growth‐enabled reprogrammable self‐folding origami. (a) Left: A schematic of the growth mechanism involving submerging the “Initial polymer” into a “nutrient” solution for concurrent nutrient uptake, polymerization, and chain exchange, followed by washing and drying to obtain the “Grown polymer”. Right: Tensile properties of two polymers grown in two “nutrient” solutions. Bottom: Chemicals in the system involving CP‐ester, NBM, crosslinker, G2, poly(CP‐ester) and poly(NBM) chains; copolymerization of CP‐ester and NBM; cross‐metathesis‐based chain exchange. (b) Chemo‐mechanical crease definition. Growth of the “living” polymer enables self‐folding of creases. (c) Kinematics prediction of the origami crane achieved by the spherical 6*R* linkage modeling. Blue arrows (left): analytical folding paths from the developed (flat) state to the folded states. Inset (middle‐bottom): spherical 6*R* linkage at the central degree‐6 vertex of the crane. θ_1_, θ_2_, θ_3_, θ_4_, θ_5_, θ_6_: six rotational angles. Inset (middle‐top): relationship between θ_1_ and θ_2_. Image (right): a flat crane sample. (d) Cranes grown in different “nutrient” solutions. Two identical flat crane samples were individually put into “1:9” (150 min) and “8:2” solution (250 min) for growth and self‐folding. Images of the folded cranes (left) and their deformation under a 200 g load (right).

We next harness growth‐induced changes in size and mechanical properties to realize reprogrammable, self‐folding origami. The active hinge is designed as a bilayer comprising a “living” polymer layer and a passive poly(cis‐cyclooctene) (poly(COE)) layer. Fabrication details are provided in Figure S1 and the Experimental Section. Briefly, the solution containing 98 vol.% cis‐cyclooctene (COE), 1 vol.% CP‐ester, 1 vol.% crosslinker, and 3.5 mg/mL G2 was injected into a mold with prepositioned “living” polymer strips, followed by polymerizing at 40°C to form robust interfacial bonding. Tensile tests (Figure S2) show that the interface remains intact even after fracture of the “living” polymers. Such strong bonding is attributed to cross‐metathesis‐induced chain exchange between poly(COE) and “living” polymers, both of which are ROMP‐based polymers and contain abundant double bonds (Supporting Information, ‘Robust interfacial bonding’). This compatibility is critical to accommodate the large mismatch strains generated during growth and folding. Poly(COE) serves as the passive panel due to its high stiffness after crystallization (Young's modulus of 265 MPa, extracted from the linear region of the tensile test in Figure S4), orders of magnitude higher than that of the “living” polymer. When submerged in a “nutrient” solution, the “living” polymer grows, driving hinge bending and origami folding. By strategically positioning the “living” polymer in a structure, both upward (mountain crease) and downward (valley crease) folds can be programmed (Figure 1b). Here, “mountain” and “valley” simply distinguish the opposite folding directions.

This versatile and simple fabrication method can be applied to diverse origami designs. As a demonstration, we programmed the mountain and valley crease assignments to fold an origami crane (Figure 1c,d). The thickness of the initial plate is 650 µm throughout. At each crease, the “living” polymer was patterned with a width of 1.8 mm and a thickness of 550 µm. The kinematic folding path of the crane has been predicted through linkage analyses. For example, we model the degree‐6 vertex on the crease pattern as the spherical 6*R* linkage, panels as rigid links, and creases as revolute joints (Figure 1c). The rotational angles of the joints define the configurations that fold from the flat state to the folded state (Supporting Information, ‘Kinematic analysis of origami structures’). Furthermore, by controlling the “nutrient” solution composition, the grown creases develop different mechanical properties, enabling cranes with different load‐bearing capacities, demonstrated by their contrasting behaviors in supporting a 200 g load (Figure 1d). The quantitative control of the mechanical properties is discussed later.

### 2.2 Controlling Folding Angle of a Beam Unit Upon Growth

We next exploit the growth of “living” polymers to control the self‐folding of an origami beam unit. The growth rate is governed by both the reaction kinetics and “nutrient” transport rate, and varies with different “nutrient” solution compositions. Thin films of “living” polymer (0.5 mm thickness × 4 mm length × 4 mm width, composition: 10% of NBM, 88% CP‐ester, and 2% crosslinker) were submerged in two “nutrient” solutions at 4°C for varying durations, and subsequently transferred out of the solution and allowed to polymerize at room temperature (22°C) for 8 h (“1:9” solution) or 24 h (“8:2” solution) to ensure thorough polymerization and chain exchange. The films were submerged in the “nutrient” solution at 4°C instead of 22°C to slow the polymerization rate. Lower temperatures extend the time available for the “nutrient” solution diffusion, allowing it to penetrate deeper into the films rather than being consumed for surface growth of the “living” polymer. The samples were then treated with ethyl vinyl ether (EVE) to deactivate G2 and remove unreacted monomers, followed by drying and weighing (Figure 2a). Additional details are provided in the Experimental Section. It shows that the mass increase is faster when growing in the “1:9” solution than in “8:2” solution. This is because NBM diffuses much more slowly than CP‐ester, and thus less NBM is absorbed and incorporated into the “living” polymer under the same growth time, even though solvents are added to the “8:2” solution to facilitate NBM diffusion. It is important to note that the mass increase in the “living” polymer does not show a plateau, in contrast to the swelling‐only behavior of hydrogels in literature or the swelling‐only behavior of the “living” polymers in a “nutrient” solution without catalyst (Figure S3), which stops once the polymer chains are fully stretched. In the “living” polymer containing catalysts, however, chain‐exchange reaction, chain scission, and insertion processes continuously relax these constraints, allowing further mass uptake and continuous growth.

**FIGURE 2:**
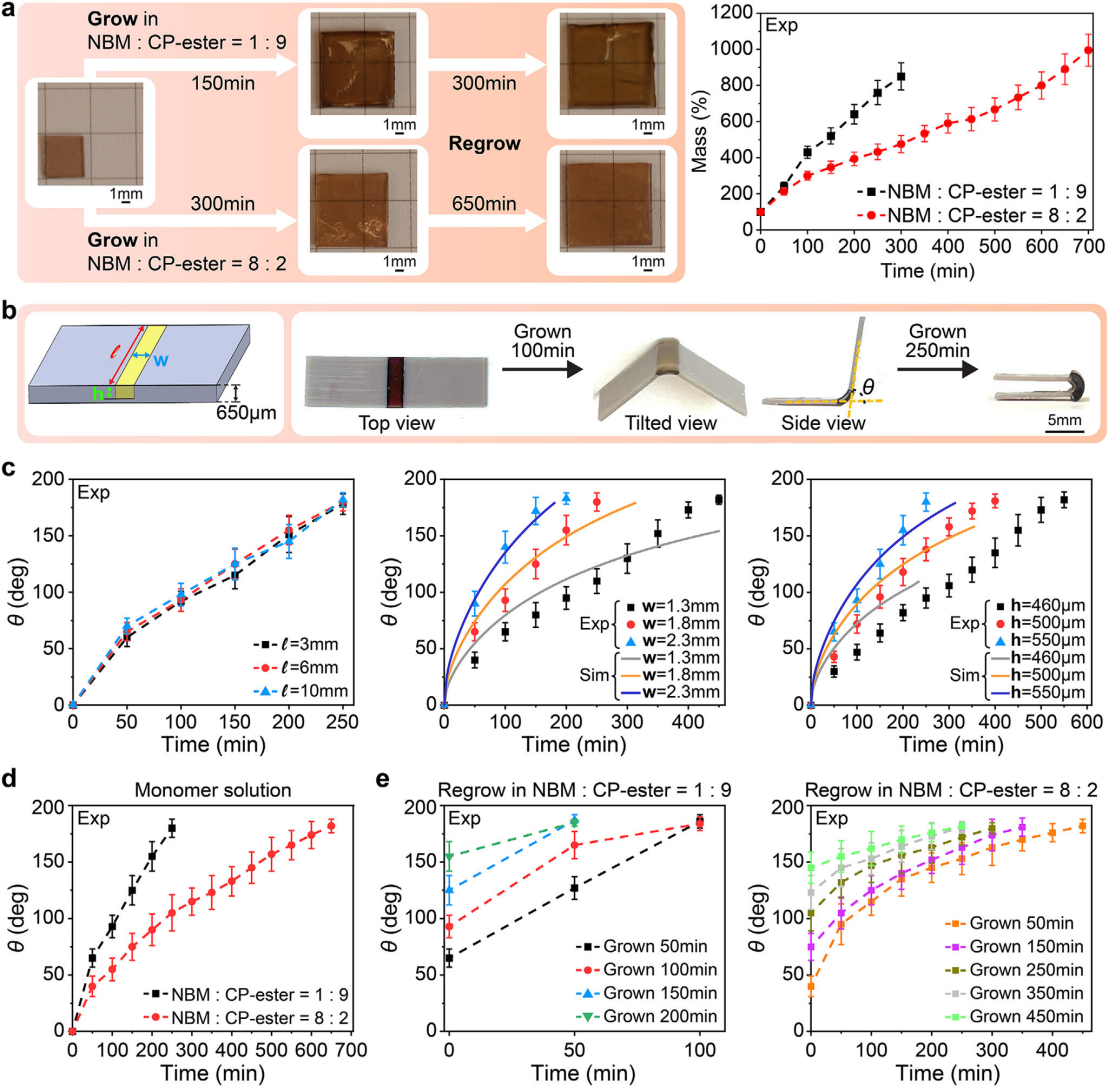
Controlling growth‐induced folding angle. (a) Left: The images of 10% NBM “living” polymers after growing in “1:9” and “8:2” solutions for different time durations. Right: The mass change of 10% NBM “living” polymers after growing in “1:9” and “8:2” solutions for varying time durations. (b) Left: Schematic illustrating the definition of “living” polymer dimensions: length (*l*), height (*h*), and width (*w*). Right: Experimental images of the beam units before and after growth for 100 and 250 min in “1:9” solution; and the definition of the folding angle *θ*. (c) Geometric influence on the folding angle: Folding angle change as a function of growth time influenced by length *l* (*w*: 1.8 mm, *h*: 550 µm) (left), width *w* (*l*: 6 mm, *h*: 550 µm) (middle), and height *h* (*l*: 6 mm, *w*: 1.8 mm) (right). Solid lines: simulation results for width and height influence. (d) Monomer composition impact: Folding angle change over growth time for different “nutrient” solution compositions: “1:9” and “8:2”. (e) Regrowth experiments: Folding angle change over regrowth time in “1:9” solution after initial growth in “1:9” for varying time durations (left), and regrowth in “8:2” solution after initial growth in “8:2” for varying time durations (right). The error bars represent the standard deviation from measuring four independent samples.

Building upon the intrinsic growth study of the “living” polymer, we next explore how growth controls the folding angles of origami beam units. Figure 2b shows the schematic and experimental results of the self‐folding of a beam unit after growth in a “1:9” “nutrient” solution for 100 and 250 min. In these experiments, the passive material is poly(COE), and the hinge is a bilayer composed of poly(COE) and the “living” polymer made of 10% NBM, 88% CP‐ester, and 2 vol.% crosslinker. The fabrication procedure followed the same steps as described previously. Beam units were immersed in the “1:9” “nutrient” solution for growth, then washed, dried, and their folding angle (*θ*, as defined in Figure 2b) was measured (details in Experimental Section). We then examined how the dimensions of the “living” polymer region affect folding angle. As shown in Figure 2c, varying the length (*l* = 3 to 10 mm) has little influence on the folding angle. As the growth time increases, the folding angle increases, and they all reach a maximum of ∼180 degrees with sufficient growth time. Increasing the width (*w*) or height (*h*) of the “living” polymer region led to larger folding angles after the same growth time (Figure 2c). Because the beam sample thickness was kept the same, increasing the height of the “living” polymer reduced the thickness of the passive poly(COE) layer, making folding easier. Numerical simulations (Figure 2c, middle and right) further confirmed the influence of geometry, predicting folding angle evolution over time that agrees well with experiments. The growth of the “living” polymer was modeled using a coupled reaction‐diffusion‐deformation framework [34], with implementation details in the Folding Angle Simulations section of the Supporting Information. Importantly, simulations considering only the species transport (i.e., swelling process) cannot reproduce the large folding angles observed (see Figure S18), highlighting the critical role of network remodeling in “living” polymers. Finally, the folding angles also vary with “nutrient” solution composition. Figure 2d shows that growth in the “1:9” solution resulted in faster folding compared to that in the “8:2” solution, consistent with the mass uptake trends in Figure 2a. Unless otherwise noted, all beam units in Figure 2c,d had an initial size of 22 mm length × 650 µm thickness.

The “living” polymers not only can keep continuously growing as long as the “nutrient” solutions are available, but can also be reactivated for additional growth after an initial growth has been halted and deactivated, enabling extended (origami) reconfiguration. To demonstrate this, films of “living” polymers (0.5 mm thickness × 4 mm length × 4 mm width) were first grown for a defined period in either “1:9” or “8:2” solutions, and then deactivated to form stable films. When these films were re‐immersed in a “nutrient” solution containing monomer, crosslinker and catalyst, they resumed growth, showing further mass increase (Figure S6). Based on this, we subjected previously folded beam units to a second round of growth (“regrowth”). For example, samples initially grown in the “1:9” solution for varying time durations were re‐submerged in the same “1:9” solution for regrowth, following the same procedure as in the first round (Figure 2e‐left). Regrowth led to a further increase in folding angles, up to 180°. Similarly, samples previously grown in the “8:2” solution also exhibited enhanced folding angles upon regrowth, reaching up to 180°. Regrowth is not limited to the original “nutrient” composition. Switching solutions (e.g., from “1:9” to “8:2”) leads to different folding kinetics and final configurations for the same starting origami structure (Figure S7 and Supporting Information, ‘Programmable regrowth via nutrient composition control’). Furthermore, regrowth is iterative and not limited to one time. As shown in Figure S8a, multiple regrowth cycles can be achieved in both “1:9” and “8:2” systems. More discussions about the versatility of regrowth are shown in the Supporting Information, ‘Iterative growth with stable shape evolution’.

### 2.3 Modulating Mechanical Properties by Growth

The mechanical properties of “living” polymers can be tailored through the growth process by adjusting the “nutrient” solution composition and growth time, providing a simple method to modulate the mechanical properties of the “living” polymers and rotational stiffness of origami creases. To characterize these changes, we measured the intrinsic tensile properties of the “living” polymers grown in “1:9” and “8:2” “nutrient” solutions for varying durations (Figure S9). The Young's modulus, extracted from the linear region of the tensile tests, is plotted against growth time in Figure 3a. It shows that the Young's modulus of the “living” polymers grown in the “1:9” “nutrient” solution remains nearly constant over 300 min of growth. This is attributed to the same chemical composition of the original polymer and the “nutrient” solution, and confirms the efficient chain exchange in the polymer system. Otherwise, significant stiffening would be expected, as it forms double network topology [35]. In contrast, growth in the “8:2” “nutrient” solution results in a gradual increase in Young's modulus of the polymer and eventually reaching a plateau (Figure 3a, Figure S9). The stiffening arises from the higher NBM:CP‐ester ratio, as the bulky ring structure of NBM restricts chain mobility and produces a more rigid polymer network. The maximum Young's modulus achieved with “8:2” solution (∼45 MPa) is approximately 45 times greater than that with “1:9” solution, demonstrating that “nutrient” solution composition and growth duration provide effective means to modulate mechanical properties of the “living” polymers over a wide range. This growth‐based tuning mechanism is general and could be extended to incorporate other ring opening metathesis monomers [36, 37], further broadening the accessible range of mechanical property changes.

**FIGURE 3:**
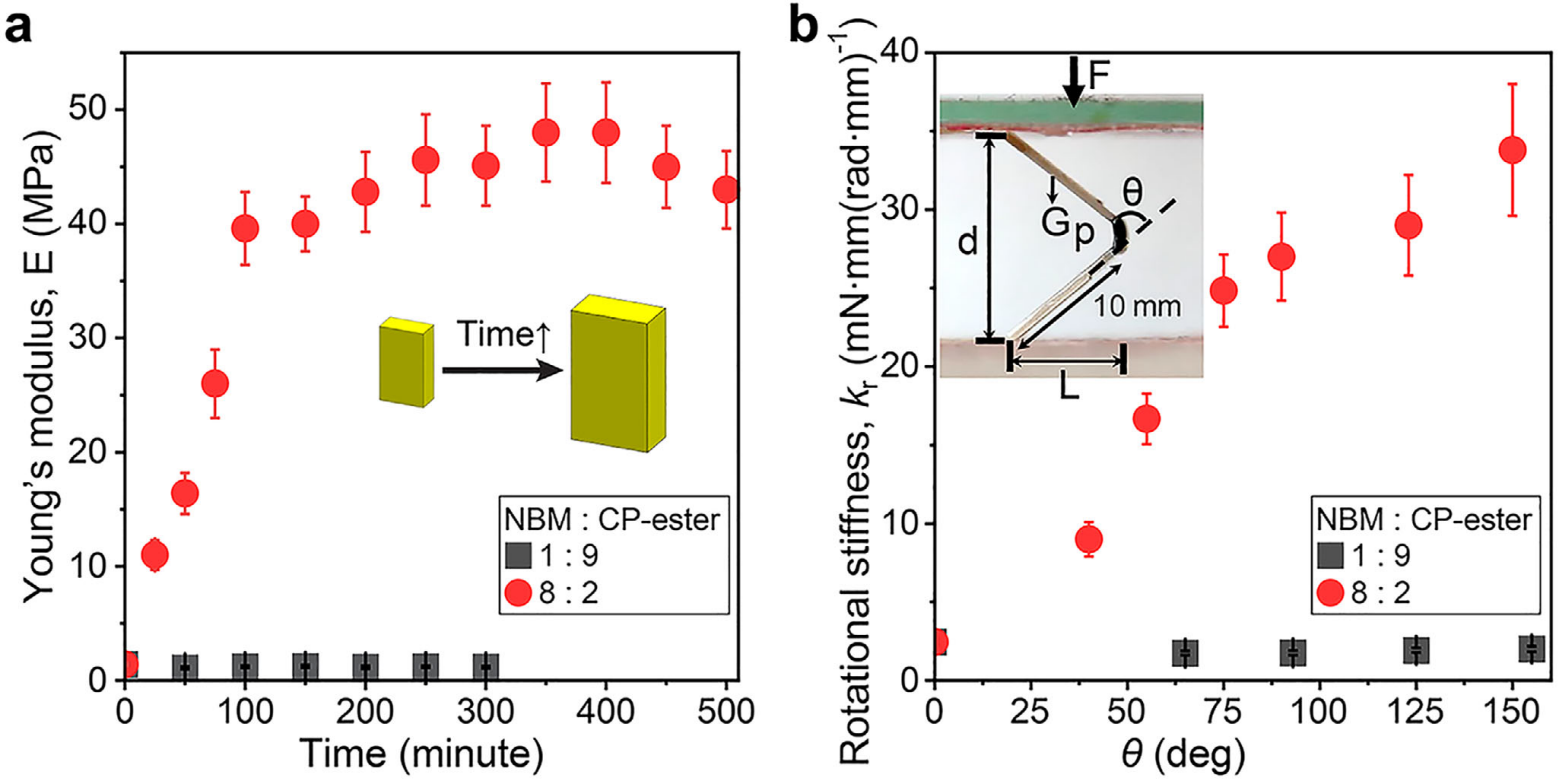
Growth‐induced mechanical property change. (a) Young's modulus of the “living” polymer versus growth time when growing in “1:9” solution and “8:2” “nutrient” solutions. (b) The rotational stiffness per unit width *k_r_* for samples grown to a certain folding angle θ in “1:9” solution and “8:2” solutions. The inset shows the experimental setup, with the poly(COE) panel near the crease being 10 mm length × 650 µm thickness × 6 mm width. The beam unit before growth has a 21.8 mm length × 650 µm thickness × 6 mm width. The error bars represent the standard deviation from measuring four independent samples.

Building upon this understanding of growth‐induced changes in intrinsic polymer properties, we next examine how it affects the mechanical behaviors of creases in origami beam units. The rotational stiffness of the crease is measured using the setup shown in the inset of Figure 3b and Figure S12a. From the compression measurement, the moment M (*M*  =  *FL* + *G_p_L*/2) as a function of angular change Δθ(Δθ  =  π − 2tan ^−1^(*d*/(2*L*)) − θ_0_) was calculated (Figure S12b,c). Here, *F* is the measured force, *G*_p_ represents the gravity of the folded unit in half, *L* denotes the horizontal length of the rigid sheet, *d* is the vertical distance between the two plates, and θ_0_ is the initial rotational angle of the sample. The initial rotational stiffness per unit width *k*_r_ ( *k*_r_ =  Δ*M*/(Δθ · *w*), where *w* is the crease length) is determined. Note that the rotational stiffness *k*_r_ refers to the structural stiffness of the hinge composed of both active “living” polymer and passive poly(COE) layers. As shown, the rotational stiffness per unit width, *k*_r_ *_,_* remains similar after growth in “1:9” solution. Moreover, growth in the “8:2” solution leads to a gradual increase in rotational stiffness, which is attributed to the increase in both the size and Young's modulus of the “living” polymer at the crease. Notably, the maximum rotational stiffness achieved with the “8:2” solution (∼32 mN·mm (rad·mm)^−1^) is 16 times larger than that with “1:9” solution (∼2 mN·mm (rad·mm)^−1^), highlighting the control of origami mechanical properties through “nutrient” solution composition.

### 2.4 Growth‐Induced Self‐Folding of Miura Origami

Building upon the understanding of single‐beam units, we extend the investigation to Miura‐ori origami tessellations. This step aims to showcase the versatility of the “living” polymer‐based fabrication method in creating more complex, repeating structures with broad application potential. Miura‐ori, a specific type of origami pattern, is known for its unique folding characteristics and has found applications in diverse fields, including aerospace [38], radiofrequency electronics [39], medical devices [40], and metamaterials [41]. Figure 4a(left) illustrates the design and growth‐induced reconfiguration of a single Miura‐ori unit, while Figure 4a(right) presents the experimental results, confirming successful folding for units with varying geometric parameters α. We observed that regardless of the α value, all units consistently achieved a dihedral angle *β* of approximately 125° after 150 min of growth. This uniformity in folding behavior highlights the robustness of our approach. Furthermore, we reprogrammed the folding angles by subjecting the units to regrowth for 150 min, resulting in a further decrease in *β*. We then progress to fabricate a 3 × 3 Miura‐ori tessellation with *α* = 15° (Figure 4b). The distribution of mountain and valley creases, which is crucial for the Miura‐ori's folding behavior, is detailed in Figure S13a. Based on the study shown in Figure 2, when growing in different “nutrient” solutions, the same tessellation design can be folded to similar angles by controlling the growth and regrowth time (Figure 4b). To assess the mechanical properties of the folded Miura‐ori tessellations, we conducted tensile tests using the setup shown in Figure 4c and Figure S13b. During these tests, the Miura‐ori tessellation exhibits horizontal expansion, which is accommodated by the Saint‐Venant fixture [42]. This customized fixture involves sliders attached to the origami sample by fish wires, allowing the sample to slide along rails with minimal friction (Figure 4c). Figure 4d presents the force‐displacement curves obtained from samples grown in “1:9” and “8:2” solutions after regrowth in Figure 4b. The results reveal significant differences in mechanical behavior between the two samples grown in “1:9” and “8:2” solutions, following the same trend as single unit's mechanical properties in Figure 3.

**FIGURE 4:**
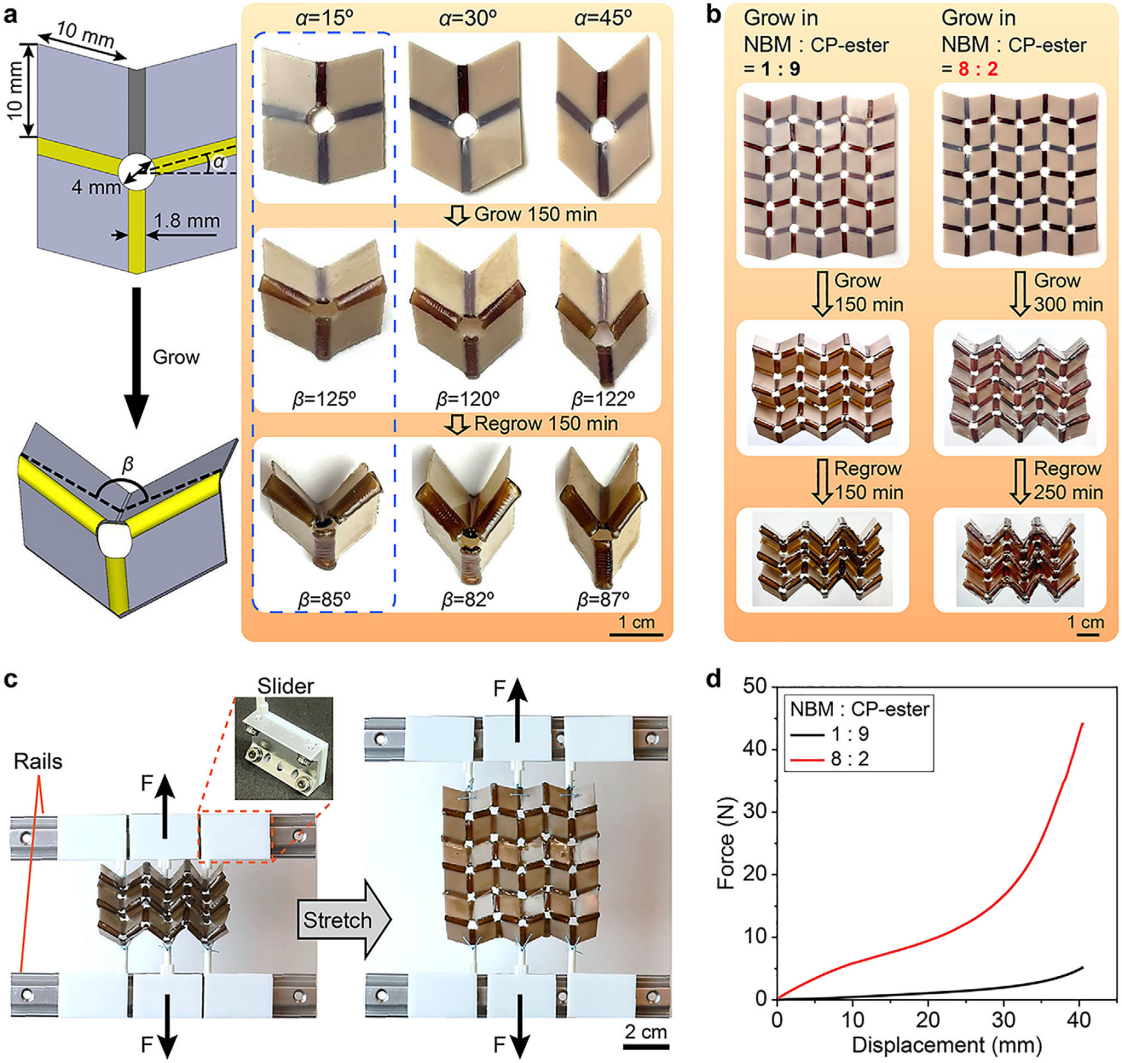
Growth‐induced self‐folding of Miura origami. (a) Left: The schematic of the single unit of Miura origami and the self‐folded structure after growth. Right: The growth and regrowth of the single units of Miura origami structures with different angle α in “1:9” “nutrient” solution. (b) The growth and regrowth induced folding of a Miura origami in “1:9” solution (left) and “8:2” solution (right). (c) The experimental setup for the tensile test of the Miura origami. (d) The tensile properties of the two folded tessellations after regrowth in b sub‐part of this figure. The “living” polymers at creases have 1.8 mm width × 550 µm thickness. All the structures have 650 µm initial thickness.

### 2.5 Growth‐Induced Self‐folding of Square‐Twist Origami

The generality of the growth‐induced self‐folding method is further demonstrated through the fabrication and folding of rigid and non‐rigid square‐twist patterns [43]. Figure 5a illustrates a square‐twist origami pattern consisting of four 4‐crease vertices with a defined mountain‐valley crease assignment. We model each vertex as a spherical 4*R* linkage to predict the kinematic folding path of the square‐twist origami. The modeling results demonstrate the relationship between the two rotational angles θ_1_ and θ_2_ during the folding process (Figure 5a: bottom‐right). Each data point on the curve corresponds to a deformed state of the square‐twist origami (e.g., see four representative states labeled as i, ii, iii, and iv). The folded state iv matches well with its physical realization (testing sample) shown as configuration #3 in Figure 5b (bottom‐left). There are in total four square‐twist configurations grown in the same “nutrient” solution with four different mountain‐valley crease assignments (Figure 5b). Two of these crease assignments are related to two rigid configurations, while the other two correspond to two non‐rigid configurations. The rigid or non‐rigid foldability is determined analytically through verification of rotational angle compatibility (Supporting Information, ‘Kinematic analysis of origami structures’, ‘Square‐twist’). We conducted tensile tests on all four configurations, i.e., two rigid and two non‐rigid (Supporting Information, ‘Kinematic prediction of folding and unfolding processes’ and Figure S15). Their mechanical responses are distinct, as shown in Figure 5c. The two non‐rigid configurations show snapping behavior along the displacement‐force curves, which is related to panel buckling phenomenon. The two rigid configurations demonstrate a monotonic displacement‐force response, as expected. Representative states of each configuration during the mechanical test are shown in Figure 5c (right). Here, the square‐twist origami demonstrations show that the growth‐induced self‐folding strategy with “living” polymers can be broadly applied to both rigid and non‐rigid origami patterns, enabling controllable reconfiguration and mechanical property tuning.

**FIGURE 5:**
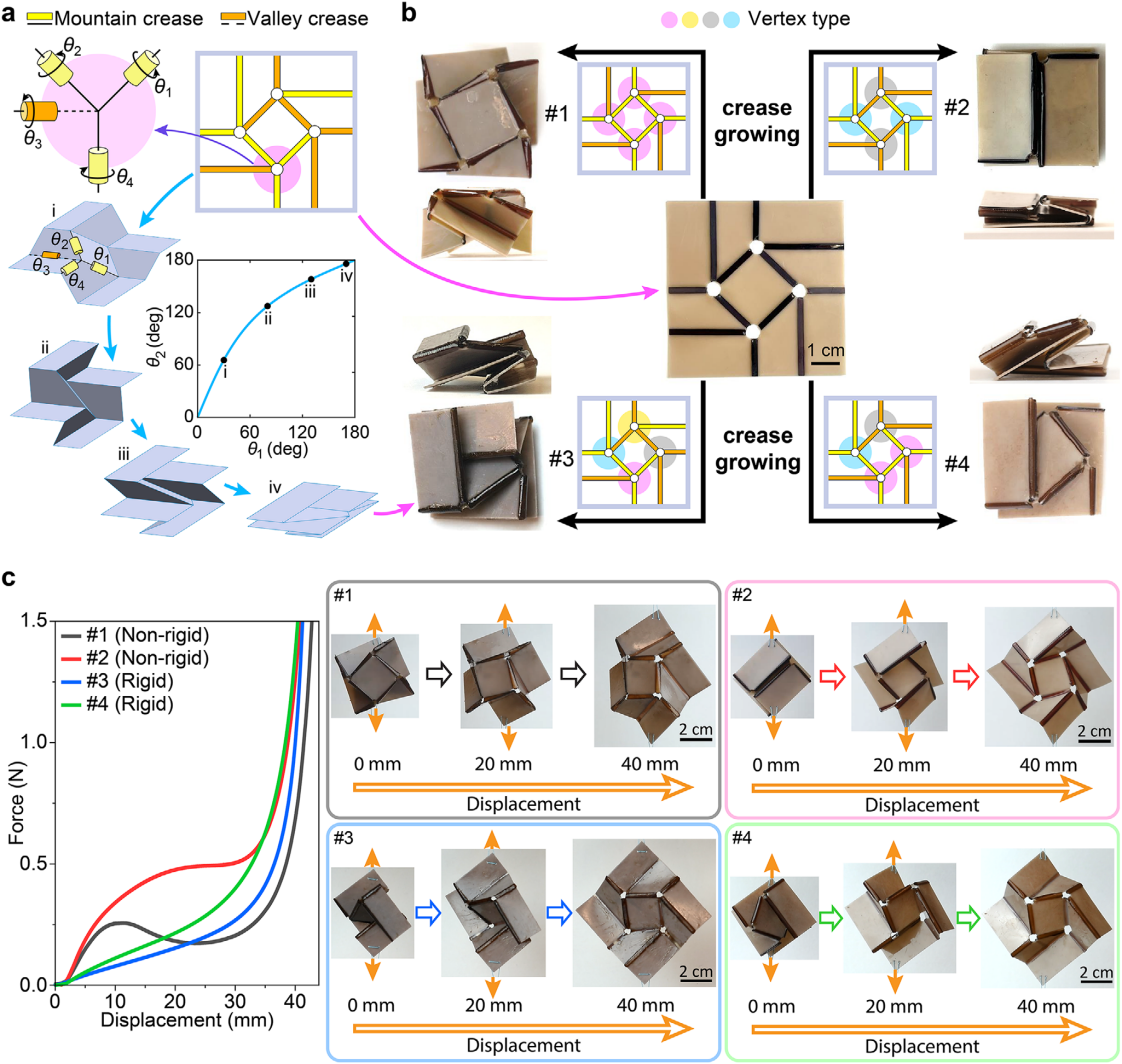
Square‐twist configurations grown in the same “nutrient” solution with different crease assignments. (a) Kinematics prediction of the square‐twist origami (top‐right) achieved by the spherical 4*R* linkages modeling (top‐left). θ_1_, θ_2_, θ_3_, θ_4_: four rotational angles at a single vertex. Blue arrows (bottom‐left) indicate the analytical folding path from the developed state to the flat‐folded state. The inset (bottom‐right) shows the relationship between the two rotational angles θ_1_ and θ_2_. (b) One square‐twist pattern (center) grows into four different configurations (corners) in the same “nutrient” solution with different mountain‐valley crease assignments. Each corner displays the schematic of the crease assignment, along with photos of the top view and side view for the self‐folded sample, respectively. (c) Tensile testing of the four folded configurations. Left, measured load‐displacement curves for each configuration. Right, each block shows three representative states filmed from the front view for each configuration. The “living” polymers at creases have a 1.8 mm width × 550 µm thickness. All the structures have 650 µm initial thickness.

## 3 Conclusions

We have developed a growth‐enabled self‐folding origami system that offers in situ control over structural reconfiguration and mechanical property tuning. By utilizing a “living” polymer capable of dynamic growth and chain remodeling, we have demonstrated precise control over folding angles and mechanical stiffness in both single‐unit beams and various rigid and non‐rigid origami tessellations. The ability to iteratively reprogram origami structures through growth and regrowth processes, coupled with the significant tunability of mechanical properties, highlights the potential of this approach. Future research will explore the integration of this system into user‐specified origami designs and investigate its application in areas such as growable origami soft robots and biomedical devices. Expanding the library of “nutrient” solutions and growth conditions may further broaden the accessible range of mechanical properties. In summary, integrating “living” polymers with origami principles establishes a fabrication platform of virtually unlimited reconfigurability and tunability—opening the door to adaptive devices that can evolve in response to engineering demands.

## 4 Experimental Section

### 4.1 Materials

All chemicals used were purchased from Fischer Scientific, if not mentioned separately, including Grubbs G2 (G2), HeatMet SIPr, K_2_CO_3_, dichloromethane (DCM), ethyl acetate (EtOAc), chloroform (CHCl_3_), acetone, ethanol, tetrahydrofuran (THF), cis‐cyclopentene (COE). 3‐Cyclopentenecarboxylic acid and methyl cyclopent‐3‐ene‐1‐carboxylate (CP‐ester) were purchased from Chemscene. Crosslinker 1,4‐phenylenebis(methylene) bis(cyclopent‐3‐ene‐1‐carboxylate) (crosslinker) was synthesized following the literature [44]. 5‐Norbornene‐2‐methanol (NBM) was purchased from Henan Fengda Chemical Co. CDCl_3_ (99.5%) was purchased from Millipore Sigma.

### 4.2 “Living” Polymer Preparation

For example, a mixture of 10 vol.% NBM, 88 vol.% CP‐ester, 2 vol.% crosslinker, and 10 mg/mL G2 was injected into a mold made of two glass plates and a rubber spacer to polymerize for 5.5 h at room temperature. The polymerized film was then demolded and put into a solution with 10 vol.% ethyl vinyl ether (EVE) and 90 vol.% DCM to quench G2 and wash off unreacted monomers, followed by drying slowly with the sample partly covered by a petri dish on a bench for 24 h, and then used for preparing origami. “Living” polymers of other NBM:CP‐ester ratios were prepared under a similar procedure, with the difference being that the time of polymerization in the mold is longer for higher NBM:CP‐ester ratios (3 h for no NBM, 7 h for 30% NBM, and 24 h for 50% NBM, 80% NBM, and 98% NBM). The mechanical property of the dried “living” polymer was measured with Instron under tension at a rate of 100% strain per minute.

### 4.3 Growth of “Living” Polymers

Multiple pieces of 10% NBM “living” polymer (made of 10 vol.% NBM, 88 vol.% CP‐ester, 2 vol.% crosslinker) in 0.5 mm thickness × 4 mm width × 4 mm length (for measuring mass) or 0.5 mm thickness × 3 mm width × 15 mm length (for measuring mechanical properties) were put into “1:9” solution (10 vol.% NBM, 88 vol.% CP‐ester, 2 vol.% crosslinker, 8 mg/mL G2) or “8:2” solution (40 vol.% NBM, 9 vol.% CP‐ester, 30 vol.% THF, 20 vol.% CHCl_3_, 1 vol.% crosslinker, 8 mg/mL G2) at 4°C for growth. Solvents THF and CHCl_3_ were added as a part of the “8:2” solution to enhance the swelling of NBM and CP‐ester into the “living” polymer, after exploring the swelling capability of various solvents into the “living” polymer (Figure S5). The “1:9” and “8:2” monomer solutions were replaced with freshly prepared ones after every ∼70 and ∼120 min, respectively. After every 50 min, one piece of the film was transferred into a petri dish and polymerized for 8 h (for “1:9”) or 24 h (for “8:2”) at room temperature, protected with argon. The samples grown in “1:9” solution were covered with glass slides during polymerization, whereas the samples grown in “8:2” were not covered with glass slides during polymerization to facilitate the evaporation of THF and CHCl_3_ out of the samples. Note that the samples growing in “8:2” were put on the bench top to evaporate THF and CHCl_3_ for 10 min after every 100 min of swelling and growth in monomer solution. The samples were then put into a solution with DCM (90 vol.%) and EVE (10 vol.%) to deactivate the catalyst, wash off unreacted monomers, and dried for 24 h. The dried “living” polymer was measured for mass and then tested with an Instron universal tensile tester model 5944 with a 1 kN load cell under uniaxial tension with 100% strain per minute to get the mechanical properties. For regrowth, the samples grown for a certain time were put into the monomer solution to grow under the same procedure as growth. Each growth experiment was repeated four times to calculate the average mass change.

### 4.4 Preparation of Single Beam Unit and Origami Structures

A 10% NBM “living” polymer (consisting of 10 vol.% NBM, 88 vol.% CP‐ester, 2 vol.% crosslinker) film was cut into strips of a certain size with a blade or laser cut into Miura origami creases for preparing origami. The “living” polymer was put into a solution of DCM with HeatMet SIPr (5 mg/mL, a ROMP catalyst used for facilitating tough bonding between poly(COE) and living polymer) for 1 min, and then put on the bench to evaporate DCM for 4 h. The 10% NBM “living” polymer strips were then compressed onto a glass plate with careful removal of gas bubbles between the polymer and the glass plate, wherein the polymer stuck to the glass plate through physical interaction, as shown in Figure S1. A 650 µm rubber spacer and a second glass plate were then placed on top of the first glass plate containing the “living” polymer to form a mold. A solution of 98 vol.% COE, 1 vol.% CP‐ester, 1 vol.% crosslinker, and 3.5 mg/mL G2 (all components were precooled to −20°C before mixing) was injected into the mold. The mold was then tightly clamped and put into a water bath at 40°C to react for 2 h. Afterward, the mold was kept on the bench to react for 8 h, followed by detaching the sample from the glass plates and reacting for 24 h in air on the bench. The films were then demolded and stored on a bench for further experiments. To ensure tough interfacial bonding between “living” polymer and poly(COE), it was found important to infuse catalyst HeatMet SIPr onto the surface of “living” polymer, and the polymerization of COE should happen at 40°C to facilitate the chain exchange between poly(CP‐ester) and poly(COE) by cross‐metathesis. Note that for preparing Miura origami, origami “crane”, and square‐twist origami structures, the “Mountain” and “Valley” creases are separately adhered to the top and bottom of the two glass plates of the mold. The crane shown in Figure 1d was made with “living” polymers at each crease measuring 1.8 mm in width and 550 µm in thickness. The total thickness of the “living” polymer and poly(COE) is 650 µm.

### 4.5 Origami Folding by Growth and Reprogramming Folding Angles by Regrowth

For measuring the growth‐induced folding angle, multiple samples of the beam unit were put into the “1:9” solution (10 vol.% NBM, 88 vol.% CP‐ester, 2 vol.% crosslinker, and 8 mg/mL G2) or “8:2” solution (40 vol.% NBM, 9 vol.% CP‐ester, 30 vol.% THF, 20 vol.% CHCl_3_, 1 vol.% crosslinker, and 8 mg/mL G2) at 4°C for growth. The “1:9” and “8:2” monomer solutions were replaced with freshly prepared ones after every 70 and 120 min, respectively. After every 50 min, one beam unit was transferred inside a petri dish and polymerized for 8 h (for “1:9”) or 24 h (for “8:2”) at room temperature, protected with argon. Note that 2 mL of CP‐ester in an open centrifuge tube was put inside the petri dish to mitigate the evaporation of CP‐ester on the swollen origami structure during polymerization. The samples growing in “8:2” solution were put on a bench top to evaporate THF and CHCl_3_ for 10 min after every 100 min of swelling and growth in the monomer solution. After polymerization, the samples were put into pure EVE for 10 min, followed by drying on a bench in a fume hood for 1 day and then measuring the folding angle with a protractor. For regrowth in “1:9” solution, beam units previously grown for certain time durations were put into “1:9” solution to grow for a certain time, following the same procedure as the first round of growth. For regrowth in “8:2” solution, the previously grown samples were first put into a solution (40 vol.% NBM, 9 vol.% CP‐ester, 30 vol.% THF, 20 vol.% CHCl_3_, and 1 vol.% crosslinker, without G2) at 50°C for 20 min. This high temperature helps the monomer solution swell into the rigid living polymer at the crease. Then, the samples were put into the “8:2” monomer solution to grow, following the same procedure as the first round of growth. The 20‐min time of swelling at 50°C is counted into the first 50 min of regrowth time. The Miura origami growth and regrowth follow the same procedure as the single‐beam unit samples.

### 4.6 Measuring Tensile Properties of the Creases

A 100 µm‐thick thin film of poly(COE) (consisting of 98 vol.% COE, 1 vol.% CP‐ester, 1 vol.% crosslinker, and 3.5 mg/mL G2) was first molded between two glass plates after 15 min of reaction at 22°C and an additional 10 min of reaction at 40°C. A film of 10% NBM “living” polymer (20 mm length × 10 mm width × varying thickness), grown in either “1:9” or “8:2” solution for a specified duration, was attached to the poly(COE) thin film. The original thickness before growth was 550 µm. The 10% NBM “living” polymer grown for a certain time was immersed into a solution of DCM with HeatMet SIPr (5 mg/mL) for 1 min, and then put on the bench to evaporate DCM for 4 h. The two layers were clapped with a clapper and a small amount of “1:9” solution was added between the poly(COE) and the “living” polymer to facilitate the bonding. The sample was then put at 40°C to react for 2 h, followed by reaction at 22°C for 24 h to form the two‐layer crease‐mimicking structure. The two‐layer structure was then measured by uniaxial tensile testing at a speed of 100% strain per minute. Additionally, the samples with different poly(COE) layer thicknesses, while maintaining a constant total thickness of 650 µm were prepared as follows. A 10% NBM “living” polymer of a certain thickness was immersed into a solution of DCM with HeatMet SIPr (5 mg/mL) for 1 min, and then put on the bench to evaporate DCM for 4 h. The “living” polymer was then placed inside a glass mold with a 650 µm rubber spacer. The COE solution (composed of 98 vol.% COE, 1 vol.% CP‐ester, 1 vol.% crosslinker, 3.5 mg/mL G2, all components precooled at −20°C before mixing) was then injected into the mold and polymerized at 40 °C for 2 h. Afterward, the mold was put on a bench to react for 8 h, followed by detaching the sample from the glass plates and reacting for 24 h in air on the bench. The films were then demolded and stored on a bench for further experiments.

### 4.7 Measuring the Rotational Stiffness of a Single Beam Unit

The single beam unit consists of two 10‐mm‐long rigid sheets connected by a “living” polymer crease at the center. The outer edges of the sheets were attached to the top and bottom plates of the testing machine using double‐sided tape. Scotch 234 tape was inserted between the sheets and the plates to allow the sample to rotate freely along the attachment edges. The experimental setup is shown in Figure S12a. Compression tests were performed at a loading speed of 30 mm/min. The measured force–displacement data were then converted into the moment–angle relationship using *M* = *FL*+*G_p_L*/2 and Δθ (Δθ  =  π − 2tan ^−1^(*d*/(2*L*)) − θ_0_, where *F* is the measured force, *G_p_* represents the gravity of the folded unit in half, *L* denotes the horizontal length of the rigid sheet, *d* is the vertical distance between the two plates, and θ_0_ is the initial rotational angle of the sample. The rotational stiffness per unit width *k_r_* ( *k_r_* =  Δ*M*/(Δθ · *w*), *w* is the crease length) was determined through linear regression for the first 20 degrees of Δθ deformation.

### 4.8 Measuring the Mechanical Properties of Miura Origami and Square‐Twist Origami

The Miura origami (3 × 3 units) was first pierced with a needle to get holes (0.635 mm diameter) on the poly(COE) panel. The distribution of the holes is demonstrated in Figure S13b. A fish line was then used to link the Miura origami with the slider through the holes, and tested under tension (30 mm/min) to measure the tensile properties. The sliders and the rails were customized and made by MiSUMi corporation. The tensile properties of square‐twist origami structures were measured in a similar way, by linking the sample to the tensile machine with fish wires (Figure 5).

## Author Contributions

Conceptualization: J.H., T.Z., G.P., Y.H. Methodology: J.H., T.Z., S.Z., D.M., H.J., Y.H., G.P. Investigation: J.H., T.Z., S.Z., D.M., H.J. Visualization: J.H., T.Z., S.Z. Funding acquisition: G.P., Y.H. Project administration: G.P., Y.H. Supervision: T.Z., G.P., Y.H. Writing – original draft: J.H., T.Z., S.Z., D.M. Writing – review & editing: J.H., T.Z., S.Z., D.M., G.P., Y.H.

## Conflicts of Interest

The authors declare no conflicts of interest.

## Supporting information




**Supporting File**: adma74348‐sup‐0001‐SuppMat.pdf.

## Data Availability

The data that support the findings of this study are available from the corresponding author upon reasonable request.
