# Supplementary material for: Autonomous Folding of Soft Matter From Living Polymers
Source: Adv Mater. 2026 Aug 29;38(60):e74348. doi: 10.1002/adma.74348 (PMC13712193; doi:10.1002/adma.74348)
Supplement: Supplementary file 1 [file ADMA-38-e74348-s001.pdf]

Supporting Information for

Autonomous Folding of Soft Matter from Living Polymers

Jiahe Huang, Tuo Zhao, Shixi Zang, Deep Malu, Huajian Ji, Glaucio H. Paulino, Yuhang Hu
Corresponding authors: gpaulino@princeton.edu, yhu462@gatech.edu

The PDF file includes:

Supplementary Text
Figs. S1 to S18
Table S1
References

Supplementary Text

Robust interfacial bonding: The “living” polymer and passive poly(COE) bonded strongly at the creases throughout growth and regrowth in all our experiments, which is supported by the tough interfacial bonding as shown in Fig. S2. In Fig. S2, “living” polymer films were bonded to poly(COE) following a similar procedure as shown in the section “Preparation of single beam unit and origami structures” in Experimental Section in the main text. The only difference is the mold used to polymerize the COE solution. Specifically, the “living” polymer films (0.5 mm thickness × 50 mm width × 40 mm length) were clamped between two glass plates surrounded by a rubber spacer (with a cavity of 0.5mm thickness × 50 mm width × 80 mm length inside the spacer), leaving only the 0.5 mm thickness × 50 mm width cross-sectional surface exposed to be bonded with COE. Then, a COE solution (98 vol.% COE, 1 vol.% CP-ester, 1 vol.% crosslinker, 3.5 mg/mL G2) was added to the mold in contact with the exposed cross-sectional surface of the “living” polymer films, and the COE solution was then polymerized to achieve tough interfacial bonding. After demolding, the sample was stretched at a rate of 6 mm/min, as shown in Fig. S2. For the two samples, both with and without a prescribed notch, the interface between poly(COE) and “living” polymer bonded firmly during the stretching process, with the fracture happening on the “living” polymers instead of the interface.

Programmable regrowth via nutrient composition control: Regrowth is not limited to the same “nutrient” solution. During each growth step, the composition of the “nutrient” solution can be adjusted. A hinge that was initially stiff can be grown into a softer one, and vice versa. Therefore, both the mechanical properties and growth kinematics of the structure can be modified after the initial fabrication. To further clarify this capability, we have conducted an experiment demonstrating that both the chemical composition and the growth sequence can be altered post-fabrication, as shown in Fig. S7. In the experiment shown in Fig. S7a, the beam used for growth contains two different “living” polymer compositions at the two creases, i.e., NBM:CP-ester = 5:5 and 1:9, wherein 5:5 represents a composition of 50% NBM, 48% CP-ester, 2% crosslinker, and 1:9 represents a composition of 10% NBM, 88% CP-ester, 2% crosslinker. Fig. S7a shows the beam’s reconfigurations upon growing in the solution containing 10% NBM, 88% CP-ester, 2% crosslinker, 8 mg/mL G2 (denoted as “1:9”) for 50 min, followed by a regrowth of the structure for another 50 min in the “1:9” solution. The difference in the folding angles for the two creases lies in the different transport rates of the solution into the “living” polymers, as supported by the data showing the different mass changes of the two “living” polymer films over time (Fig. S7c). The mass change in Fig. S7c was measured by immersing films of the two “living” polymers (0.5 mm thickness × 4 mm width × 4 mm length) in the “1:9” solution for growth, following the same growth procedure detailed in the “Growth of “living” polymers” section in Experimental Section. Moreover, the same starting beam changed to a different configuration when substituting the “nutrient” composition with “8:2” (40 vol.% NBM, 9 vol.% CP-ester, 30 vol.% THF, 20 vol.% $CHCl_3$, 1 vol.% crosslinker, 8 mg/mL G2) during the regrowth phase, as shown in Fig. S7b. The left crease containing a “living” polymer of 5:5 also folds up in the “8:2” “nutrient” solution, which is supported by the mass increase upon growth of the “living” polymer films shown in Fig. S7d. This simple demonstration showcases how a single starting structure can access different folded configurations through a combination of the prescribed distribution of the “living” polymers’ chemical compositions at creases and sequential regrowth in varied “nutrient” solutions.

Iterative growth with stable shape evolution: To further substantiate the iterative nature of the growth and regrowth processes, an additional experiment was conducted using beam units submerged in two distinct “nutrient” solutions, as shown in Fig. S8. The “8:2” solution contains 40 vol.% NBM, 9 vol.% CP-

ester, 30 vol.% THF, 20 vol.% CHCl3, 1 vol.% crosslinker, 8 mg/mL G2, whereas the “1:9” solution contains 10% NBM, 88% CP-ester, 2% crosslinker, and 8 mg/mL G2. These units underwent three iterations of growth, consisting of an initial growth and stop (deactivating catalyst, washing and drying the polymer) followed by two subsequent regrowth and stop, as illustrated in Fig. S8a. Each iteration constitutes 50 min of growth in the solutions following the same growth procedure shown in the “Growth of “living” polymers” section in the Experimental Section. The continuous, stepwise increase in folding angles across the three iterations demonstrates the robust capacity for repeatable regrowth. Furthermore, the folding angles achieved after the third iteration remained stable during a five-day storage period at room temperature on the bench (Fig. S8b). This stability confirms the successful chemical integration of monomers into the creases, allowing the origami to reach and maintain stable intermediate states.

Kinematic analysis of origami structures: The origami structures are composed of multiple vertices. Each vertex connects to multiple creases. We kinematically model an m-crease vertex as a spherical mR linkage to predict folding paths of the origami structures (Fig. S14a). In the spherical linkage, we represent creases as revolute joints, which are connected by rigid links. Since the spherical mR linkage is a single-loop linkage consisting of m links intersecting at one point, we present the closure equation, based on Denavit-Hartenberg (D-H) notation set-up[1], as follows:

$$\mathbf{Q}_{21} \cdot \mathbf{Q}_{32} \cdot \mathbf{Q}_{43} \cdots \mathbf{Q}_{m(m-1)} \cdot \mathbf{Q}_{1m} = \mathbf{I}_3, \tag{1}$$

where the transformation matrix $\mathbf{Q}$ is given by:

$$\mathbf{Q}_{(i+1)i} = \begin{bmatrix} \cos\theta_i & -\cos\alpha_{i(i+1)} \cdot \sin\theta_i & \sin\alpha_{i(i+1)} \cdot \sin\theta_i \\ \sin\theta_i & \cos\alpha_{i(i+1)} \cdot \cos\theta_i & -\sin\alpha_{i(i+1)} \cdot \cos\theta_i \\ 0 & \sin\alpha_{i(i+1)} & \cos\alpha_{i(i+1)} \end{bmatrix}, \tag{2}$$

where the parameter θ_i (i=1, 2, …, m) is the rotation angle of the ith joint, and $\alpha_{i(i+1)}$ is the sector angle between the ith and $(i+1)^{th}$ joints. When $i = m$, the term $(i+1)$ is replaced by 1. Moreover, we derive the relationship between the rotational angles by substituting Eq. (2) into Eq. (1). The rotating direction of the joint is defined in a right-handed system, and its rotational angle θ ranges from $-\pi$ to π. Here, we define clockwise rotation as positive (red arrow), while counterclockwise rotation is negative. As shown in Fig. S14b, the rotation of the joint relates to the definition of the corresponding crease: valley crease or mountain crease. For the valley crease, the joint rotates in a counterclockwise direction, and the rotational angle θ ranges from $-\pi$ to 0. Then, the dihedral angle β of the valley crease is calculated by $\beta = \pi + \theta$. For the mountain crease, the joint rotates in a clockwise direction, and the rotational angle θ ranges from 0 to π. Thus, the dihedral angle β of the mountain crease is calculated by $\beta = \pi - \theta$. In the following sections, we present detailed rotational angle formulas in the following sections using spherical 4R and 6R linkages as examples corresponding to four-crease and six-crease vertices, respectively.

Spherical 4*R* linkage: For the spherical 4*R* linkage, we rewrite Eq. (1) as:

$$\mathbf{Q}_{21} \cdot \mathbf{Q}_{32} \cdot \mathbf{Q}_{43} \cdot \mathbf{Q}_{14} = \mathbf{I}_3. \tag{3}$$

Substituting Eq. (2) into Eq. (3), we obtain the relationship between the rotation angles θ_i and $\theta_{(i+1)}$ as in [43]:

$$
\begin{aligned}
&\cos\alpha_{(i+1)(i+2)}\cdot\sin\alpha_{(i-1)i}\cdot\sin\alpha_{i(i+1)}\cdot\cos\theta_i\\
&+\cos\alpha_{(i-1)i}\cdot\sin\alpha_{i(i+1)}\cdot\sin\alpha_{(i+1)(i+2)}\cdot\cos\theta_{(i+1)}\\
&+\cos\alpha_{i(i+1)}\cdot\sin\alpha_{(i+1)(i+2)}\cdot\sin\alpha_{(i-1)i}\cdot\cos\theta_i\cdot\cos\theta_{(i+1)}\\
&-\sin\alpha_{(i+1)(i+2)}\cdot\sin\alpha_{(i-1)i}\cdot\sin\theta_i\cdot\sin\theta_{(i+1)}\\
&+\cos\alpha_{(i+2)(i+3)}-\cos\alpha_{i(i+1)}\cdot\cos\alpha_{(i+1)(i+2)}\cdot\cos\alpha_{(i-1)i}=0.
\end{aligned}
\tag{4}
$$

Equation (4) can be simplified using the definition of sector angles for different origami patterns.

Miura-ori: For the Miura-ori pattern (Fig. S14c), we define the sector angles as:

$$\alpha_{12}=\alpha_{23}=\alpha,\alpha_{34}=\alpha_{41}=\pi-\alpha. \tag{5}$$

Substituting Eq. (5) into Eq. (4), we obtain the solution of the kinematic relationship as:

$$\tan(\theta_2/2)=-\cos\alpha\tan(\theta_1/2)\,,\theta_3=\theta_1,\theta_4=-\theta_2. \tag{6}$$

Square-twist: For the square-twist origami pattern (Fig. S14d), we define the sector angles of each vertex as:

$$\alpha_{12}=\pi/2\,,\alpha_{23}=\alpha,\alpha_{34}=\pi/2\,,\alpha_{41}=\pi-\alpha. \tag{7}$$

Substituting Eq. (7) into Eq. (4), we obtain two solutions, which rely on different mountain-valley assignments. For the MV assignment in the pink and yellow vertices, the solution of the kinematic relationship is in [43]:

$$\tan(\theta_2/2)=\cos\alpha\tan(\theta_1/2)/(1-\sin\alpha)\,,\theta_3=-\theta_1,\theta_4=\theta_2. \tag{8}$$

For the MV assignment in the blue and grey vertices, the solution of the kinematic relationship is:

$$\tan(\theta_2/2)=-\cos\alpha\tan(\theta_1/2)/(1+\sin\alpha)\,,\theta_3=\theta_1,\theta_4=-\theta_2. \tag{9}$$

When combining four vertices (A, B, C, and D) in a sequence to create a square-twist origami pattern, the rotational angles of two vertices along the common crease have the same value, i.e.,

$$\theta_2^{\mathrm{A}}=\theta_1^{\mathrm{B}},\ \theta_2^{\mathrm{B}}=\theta_1^{\mathrm{C}},\ \theta_2^{\mathrm{C}}=\theta_1^{\mathrm{D}},\text{ and }\theta_2^{\mathrm{D}}=\theta_1^{\mathrm{A}}. \tag{10}$$

Then, we define the rigid foldability of a square-twist origami pattern through Eqs. (8)-(10). For example, in square-twist #1unit cell, we have

$$
\begin{aligned}
&\tan(\theta_2^A/2)=\cos\alpha\tan(\theta_1^A/2)/(1-\sin\alpha)\,,\theta_1^B=\theta_2^A,\\
&\tan(\theta_2^B/2)=\cos\alpha\tan(\theta_1^B/2)/(1-\sin\alpha)=\cos^2\alpha\tan(\theta_1^A/2)/(1-\sin\alpha)^2\,,\theta_1^C=\theta_2^B,\\
&\tan(\theta_2^C/2)=\cos\alpha\tan(\theta_1^C/2)/(1-\sin\alpha)=\cos^3\alpha\tan(\theta_1^A/2)/(1-\sin\alpha)^3\,,\theta_1^D=\theta_2^C,\\
&\tan(\theta_2^D/2)=\cos\alpha\tan(\theta_1^D/2)/(1-\sin\alpha)=\cos^4\alpha\tan(\theta_1^A/2)/(1-\sin\alpha)^4\,.
\end{aligned}
\tag{11}
$$

Equation (11) indicates that $\theta_2^{\mathrm{D}}\neq\theta_1^{\mathrm{A}}$, resulting in non-rigid foldability. By contrast, in square-twist #3, we have

$$
\begin{aligned}
&\tan(\theta_2^A/2)=\cos\alpha\tan(\theta_1^A/2)/(1-\sin\alpha)\,,\theta_1^B=\theta_2^A,\\
&\tan(\theta_2^B/2)=-\cos\alpha\tan(\theta_1^B/2)/(1+\sin\alpha)=-\tan(\theta_1^A/2)\,,\theta_1^C=\theta_2^B,\\
&\tan(\theta_2^C/2)=\cos\alpha\tan(\theta_1^C/2)/(1-\sin\alpha)=-\cos\alpha\tan(\theta_1^A/2)/(1-\sin\alpha)\,,\theta_1^D=\theta_2^C,\\
&\tan(\theta_2^D/2)=-\cos\alpha\tan(\theta_1^D/2)/(1+\sin\alpha)=\tan(\theta_1^A/2)\,.
\end{aligned}
\tag{12}
$$

Equation (12) indicates that $\theta_2^{\mathrm{D}} = \theta_1^{\mathrm{A}}$, resulting in rigid foldability.

Spherical 6*R* linkage: For the spherical 6*R* linkage, we rewrite Eq. (1) as:

$$\mathbf{Q}_{21} \cdot \mathbf{Q}_{32} \cdot \mathbf{Q}_{43} \cdot \mathbf{Q}_{54} \cdot \mathbf{Q}_{65} \cdot \mathbf{Q}_{16} = \mathbf{I}_3. \tag{13}$$

Since a general spherical 6*R* linkage is of three degrees of freedom, we add symmetric conditions to reduce this number and simplify the calculation of Eq. (13). According to the vertex in Fig. S14e, the symmetric conditions are given by:

$$\theta_1 = \theta_4, \theta_2 = \theta_3 = \theta_5 = \theta_6. \tag{14}$$

Additionally, we define the sector angles as:

$$\alpha_{12} = \alpha_{34} = \alpha_{45} = \alpha_{61} = \alpha, \alpha_{23} = \alpha_{56} = \pi - 2\alpha. \tag{15}$$

Substituting Eqs. (2), (14), and (15) into Eq. (13), we obtain the relationship between the rotation angles as[2]:

$$\tan(\theta_1/2) = -\cos\alpha \tan(\theta_2/2), \theta_4 = \theta_1, \theta_3 = \theta_5 = \theta_6 = \theta_2. \tag{16}$$

Kinematic prediction of folding and unfolding processes: The folding and unfolding processes of the square-twist unit cells can be predicted through the kinematic analysis[3]. The kinematic results shown in Fig. S15 are obtained using $\alpha = 44°$ for cell #1 and $\alpha = 45°$ for cells #2, #3, and #4. For the two non-rigid cells, an additional crease is introduced into the central square to account for panel deformation, and the resulting modified patterns exhibit two distinct folding paths. For cell #1, the experimentally observed unfolding process is consistent with path 2, because path 1 leads to panel intersections. For cell #2, the experimentally observed unfolding process follows path 1 before the bifurcation point and then switches to path 2 due to the principle of minimum energy[4]. For the two rigid square-twist unit cells, the unfolding processes can be accurately predicted by the kinematic analysis, since their deformation is primarily accommodated by the rotation of the creases."

Folding angle simulations

Dynamic "living" polymer growth theory: The dynamic "living" polymer is modeled based on the coupled reaction-diffusion-deformation theory in reference [34] of the main text. The theory is summarized here. Please refer to the theory paper for more details. All the listed equations are written using non-dimensional variables.

The dynamic gel system uses the polymerization reaction equilibrium and the chain exchange reactions to update the network backbone of the gel. At the same time, the coupled reaction-diffusion-deformation dynamics drive the mobile species, namely the monomers and the crosslinkers, in and out of the system. The evolving polymer backbone is described using the following three variables: the chain density C, the segment density C_n, and the chain distribution tensor $\boldsymbol{\gamma}$. The chain density is defined as the number of chains per reference volume, where a chain is any continuous polymer segment between two crosslinkers. Similarly, the segment density is defined as the number of Kuhn segments in the polymer backbone per unit reference volume. The chain distribution tensor describes the average stretch and orientation of the polymer chains in the network. Next, we consider two types of mobile species in the system: the monomer concentration C_m and the crosslinker concentration C_{cr}. These concentrations (C_m, C_{cr}) are defined as the number of species per unit reference volume. The dry polymer state is used as the reference state.

The polymerization reaction equilibrium and the chain-exchange reaction are described in terms of the following 3 first order reaction: forward reaction (rate k_f), backward reaction (rate k_b) and chain exchange reaction (rate k_d). The forward reaction converts monomers (that have swollen in the gel system) to segments (on the polymer backbone). While the backward reaction converts segments back to monomers. The polymerization reaction equilibrium is controlled by the forward to backward reaction rate ratio. Further, segments formed during the forward reaction can either create new chains or be inserted into existing chains. We use a parameter r_{new} that describes the fraction of segments formed during forward reactions that go into formation of new chains. This parameter is described in terms of a phenomenological parameter α. The evolution equations for the network variables and mass balance relations for the mobile species, due to reaction, diffusion and deformation are described as follows.

$$\frac{dC}{dt} = r_{\text{new}} k_f \frac{C_{cr}}{J} \tag{17}$$

$$\frac{dC_n}{dt} = -k_f \frac{C_m}{J} + k_b \frac{C_n}{J} \tag{18}$$

$$\frac{d\boldsymbol{\gamma}}{dt} = -\frac{2}{3} \frac{(1 - r_{new})k_f C_m - k_b C_n}{C_n J} \boldsymbol{\gamma} + \left(r_{new} \frac{f}{2} \frac{k_f C_{cr}}{CJ} + \frac{k_d}{J} \right) (\mathbf{I} - \boldsymbol{\gamma}) + \mathbf{L}\boldsymbol{\gamma} + \boldsymbol{\gamma}\mathbf{L}^{\mathrm{T}} \tag{19}$$

$$r_{new} = \frac{C_{cr}}{\alpha C + C_{cr}} \tag{20}$$

$$\frac{dC_m}{dt} + \nabla \cdot \mathbf{J}_m = k_f \frac{C_m}{J} - k_b \frac{C_n}{J} \tag{21}$$

$$\frac{dC_{cr}}{dt} + \nabla \cdot \mathbf{J}_{cr} = -\frac{2 r_{\text{new}}}{f} k_f \frac{C_{cr}}{J} \tag{22}$$

Here $\mathbf{F}$ is the deformation gradient, $J = \det \mathbf{F}$ is the swelling ratio, $\mathbf{L} = \dot{\mathbf{F}}\mathbf{F}^{-1}$ is the velocity gradient. Further, $\mathbf{J}_m$ and $\mathbf{J}_{cr}$ are the nominal fluxes for monomer and crosslinker, respectively. The nominal fluxes are defined as the number of molecules crossing the material element per unit area per unit time. These fluxes are calculated from their respective chemical potential gradients as follows:

$$\mathbf{J}_m = -D_m C_m \mathbf{F}^{-1} \mathbf{F}^{-T} \frac{\partial \mu_m}{\partial \mathbf{X}} \tag{23}$$

$$\mathbf{J}_{cr} = -D_{cr} C_{cr} \mathbf{F}^{-1} \mathbf{F}^{-T} \frac{\partial \mu_{cr}}{\partial \mathbf{X}} \tag{24}$$

Here μ_i denotes the chemical potential and D_i denotes the diffusion coefficient for specie $i (= m, cr)$. Further, the force balance equation for the gel system is as follows.

$$\nabla \cdot \mathbf{P} + \mathbf{B} = \mathbf{0} \tag{25}$$

Here $\mathbf{P}$ is the first Piola-Kirchhoff stress tensor, and $\mathbf{B}$ is the body force. The Eqs. (21), (22) and (25) are the governing equations for this system. Furthermore, the exact form of the stress tensor $\mathbf{P}$ and the chemical potentials μ_m and μ_{cr} is given by the constitutive laws derived using the Coleman-Noll procedure. It depends on the specific form of the free energy function for the system. Also, the following

incompressibility constraint is inforced on the system which states that the total volume of the gel is sum of the volumes of segments and monomers. Note that both segments and monomers are assumed incompressible and have the same volume.

$$J = C_n + C_m \tag{26}$$

The enforcement of this incompressibility constraint introduces a Lagrange multiplier, the Osmotic pressure Π. The stress tensor and chemical potentials can be calculated as follows.

$$\mathbf{P} = C(\boldsymbol{\gamma} - \mathbf{I})\mathbf{F}^{-T} - \Pi J \mathbf{F}^{-T} \tag{27}$$

$$\mu_m = \ln\frac{C_m}{J} + \frac{C_n}{J} + \frac{\chi C_n^2}{J^2} - \frac{C_{cr}}{J} + \Pi \tag{28}$$

$$\mu_{cr} = \ln\frac{C_{cr}}{J} \tag{29}$$

Where χ is the Flory-Huggins interaction parameter, describing the enthalpy of mixing between the polymer and monomer solution. This concludes the modeling theory for the active "living" gel system.

Panel material theory: We use compressible neo-Hookean material to model the passive layer in the bilayer crease structure, which is made with poly(COE). The first Piola-Kirchhoff stress tensor for the passive material is given as follows:

$$\mathbf{P} = G_P(\mathbf{F} - \mathbf{F}^{-T}) + \frac{K_P}{2}(J^2 - 1)\mathbf{F}^{-T} \tag{30}$$

Here G_P and K_P represent the shear and bulk modulus for the passive material, respectively. We ensure $K_P \gg G_P$ to enforce incompressibility.

Basic simulation procedure: In this section, we numerically study the self-folding behavior of the origami crease. The composite bilayer crease is made of an active "living" polymer layer ($l \times w \times h$) and the passive panel material layer. The total height of the bilayer crease is kept constant, $h_T = 650$ µm, as shown in Fig. 2b. Since the length of the crease (l) is large compared to the active layer width and height, the crease folding due to growth is simulated as a 2D plain strain problem (see Fig. S16). This 2D boundary value problem is solved numerically using the custom physics feature of COMSOL 6.2. The dynamic "living" gel model is applied to the active domain using the above-mentioned coupled differential equations (see Eqs. (17)-(29)). While for the passive domain, the compressible neo-Hookean material model is used (see Eq. (30)). At the interface between the active and passive domains, force and displacement continuity is ensured. The mobile species, monomer and crosslinker, can diffuse in and out of the active domain. The passive domain is impermeable to these mobile species. The effect of active material size on the total folding angle is studied.

Boundary conditions, time steps and mesh: As the folding process is symmetric along the middle axes, only the right half of the 2D rectangular geometry is modeled. Based on the active and passive domain sizes, the 2D geometry is created as shown in Fig. S16. Simulations are performed for 3 heights and 3 width combinations as shown in Fig. 2c-middle and right. The total height is kept constant at $h_T = 650$ µm, while the total length is shortened, ensuring no effect on the folding angle. Folding angle is measured from the slope of the right edge.

The monomer and crosslinker molecules can diffuse in from the top edge of the active domain. This is done by a constant chemical potential boundary condition, while any species transport is disallowed for all other interfaces/boundaries. Symmetry boundary conditions are applied on the left edge. That means no displacement along x direction ($u = 0$) and no species transport. While the bottom left vertex is fixed ($u = 0, v = 0$). Here u and v describe the displacement along x and y directions, respectively.

The simulation is performed in two steps. In the first step, the chemical potential boundary condition at the top edge of the active domain is quickly ramped up from the initial chemical potential to the external chemical potential. The initial state is supposed to be dry polymer, but to avoid numerical singularity issues, a small uniform monomer and crosslinker concentration is assumed throughout the active domain. This creates a chemical potential gradient at the top boundary and allows for species transport. During this step, the reactions are arrested (the reaction constants are kept at zero). In the second step, the constant chemical potential is held, and the reactions are started. The simulation continues until the folding angle reaches 180 degrees, that is full folding.

The 2D geometry is meshed using structured quadrilateral meshes. The mesh density is kept high at the active-passive interface and at the top boundary.

Parameter selection and fitting: Since we are currently concerned with the effect of geometry on the folding angle, we assume the backward reaction constant to be zero ($k_b = 0$). This means all the monomer/crosslinker that diffuse in the active domain are polymerized. This simplification avoids a third step in the simulation, where the reaction needs to be arrested and excess monomer/crosslinker are allowed diffuse out to completely dry the active domain. Accordingly, the adjusted forward reaction rate, chain exchange rate and diffusion coefficients are estimated from growth and free swelling experiments. The shear modulus of the passive polymer is measured from the tensile test, and the bulk modulus is assumed to be 100 times the shear modulus to simulate incompressible behavior. From the initial modulus of the active polymer, we obtain the initial chain density. The monomer diffusion coefficient is fitted from the free swelling experiments. The quantities are listed below. All quantities are normalized.

Table S1. The parameters used in the simulation

Parameter	Description	Values (normalized)
k_f	Forward reaction rate	$7E-4$
D_m	Monomer diffusion coefficient	$1.4\text{E}-10$
k_b	Backward reaction rate	0
k_d	Chain exchange rate	$0.1\ k_f$
C	Initial chain concentration	0.4
G_P	Passive domain shear modulus	40
K_P	Passive domain bulk modulus	$100\ G_P$

Fig. S17 a-f shows the various time snapshots during the simulation, for $w = 1.8$ mm and $h = 550$ μm. The contours show the volume expansion ratio variation. The full folding angle range, from initial 0° to the final 180°, can be recovered from the simulation. Further, a parametric study and experimental comparison can be seen in Fig. 2c. The simulation describes the experimental trend: for similar growth time, a larger folding angle is achieved for higher active material sizes (both height and width). The model and experiments show poorer agreement for samples with the smallest heights and widths of the active material,

as these geometries require longer growth times to achieve complete folding. At extended growth times, discrepancies between simulations and experiments arise because the current model does not fully capture the evolution of growth and stiffness at the interface. As the mismatch strain increases, elements near the interface become highly distorted, leading to premature termination of the simulation. These limitations are primarily computational and could be addressed in the future with improved solvers or adaptive meshing techniques.

Further Fig. S18a shows the folding angle change comparison between swelling only and growth systems for the $w = 1.8\ mm, h = 550\ \mu m$ geometry case. A volumetric change of more than 1 order of magnitude is seen in growth simulations. In the dynamic "living" system, the polymer network gets remodeled, which allows for large increase in size and stiffness. While a swelling only system is limited in the extent of swelling based on the maximum stretchability of the polymer chains (see Fig. S18b). Also, the modulus of the gel decreases during swelling. Hence a simulation setup that considers only swelling cannot capture the folding angle behavior we see, and further swelling based cannot describe the increase in mechanical strength of the polymer during the growth process, which is essential is estimating the hybrid-bilayer folding behavior. Therefore, a growth-based model as described in Eqs. (17)-(29) is needed for such a study.

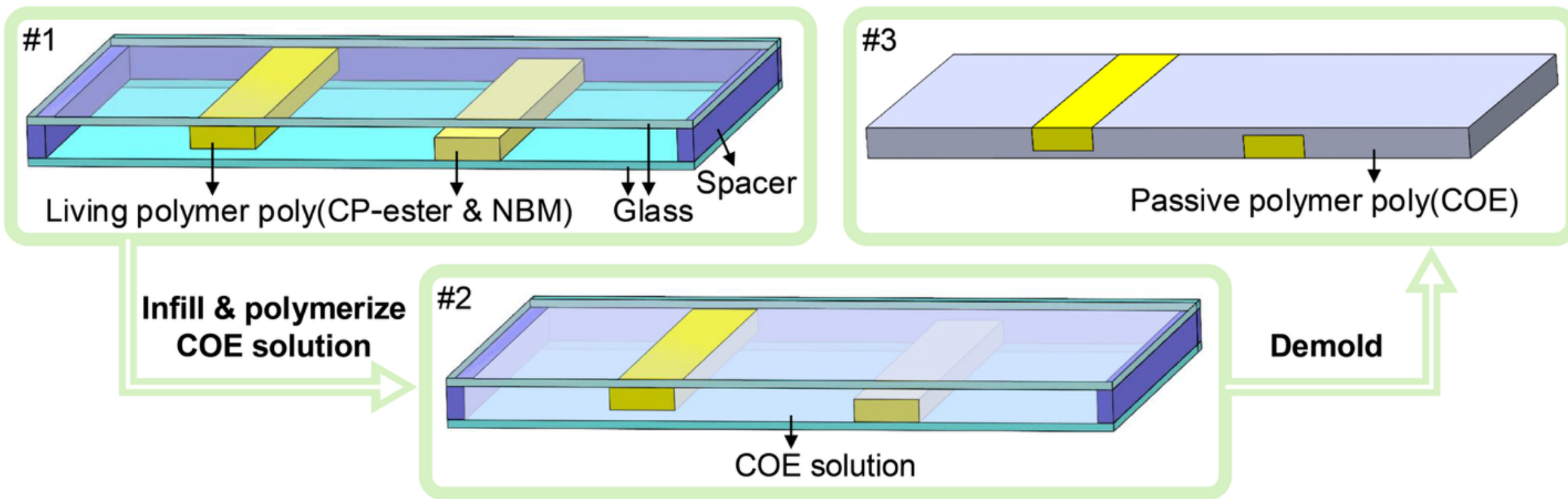

Fig. S1 The origami fabrication procedure. The COE solution is composed of 98 vol.% COE, 1 vol.% CP-ester, 1 vol.% crosslinker, and 3.5 mg/mL G2.

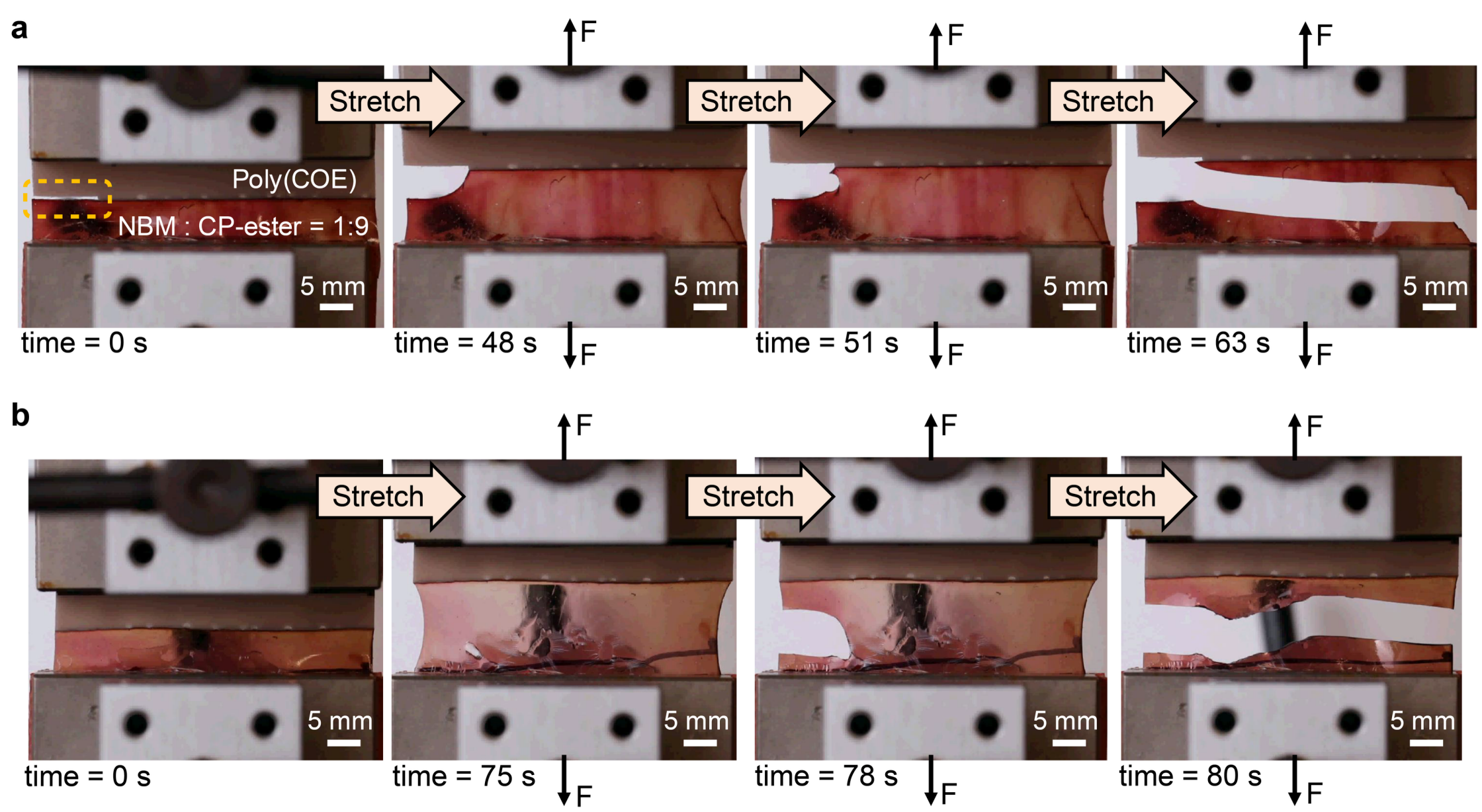

Fig. S2. **a** The images showing the sample status upon stretching. The sample consists of 0.5 mm-thick poly(COE) bonded in-plane to a 0.5 mm-thick “living” polymer composed of NBM:CP-ester = 1:9 (10% NBM, 88% CP-ester, 2% crosslinker). The sample has a prescribed notch circled, shown in a yellow dashed line. The notch was prepared by partly covering the cross-sectional surface of the “living” polymer film with a rubber spacer (0.5 mm × 10 mm × 1 mm) before bonding with poly(COE). **b** The images showing the sample without a notch being stretched. The sample consists of the same poly(COE) and “living” polymer as in **a**, but differs by not having a notch. The samples were stretched at 6 mm/min. Scale bars in Fig. S2 all represent 5 mm.

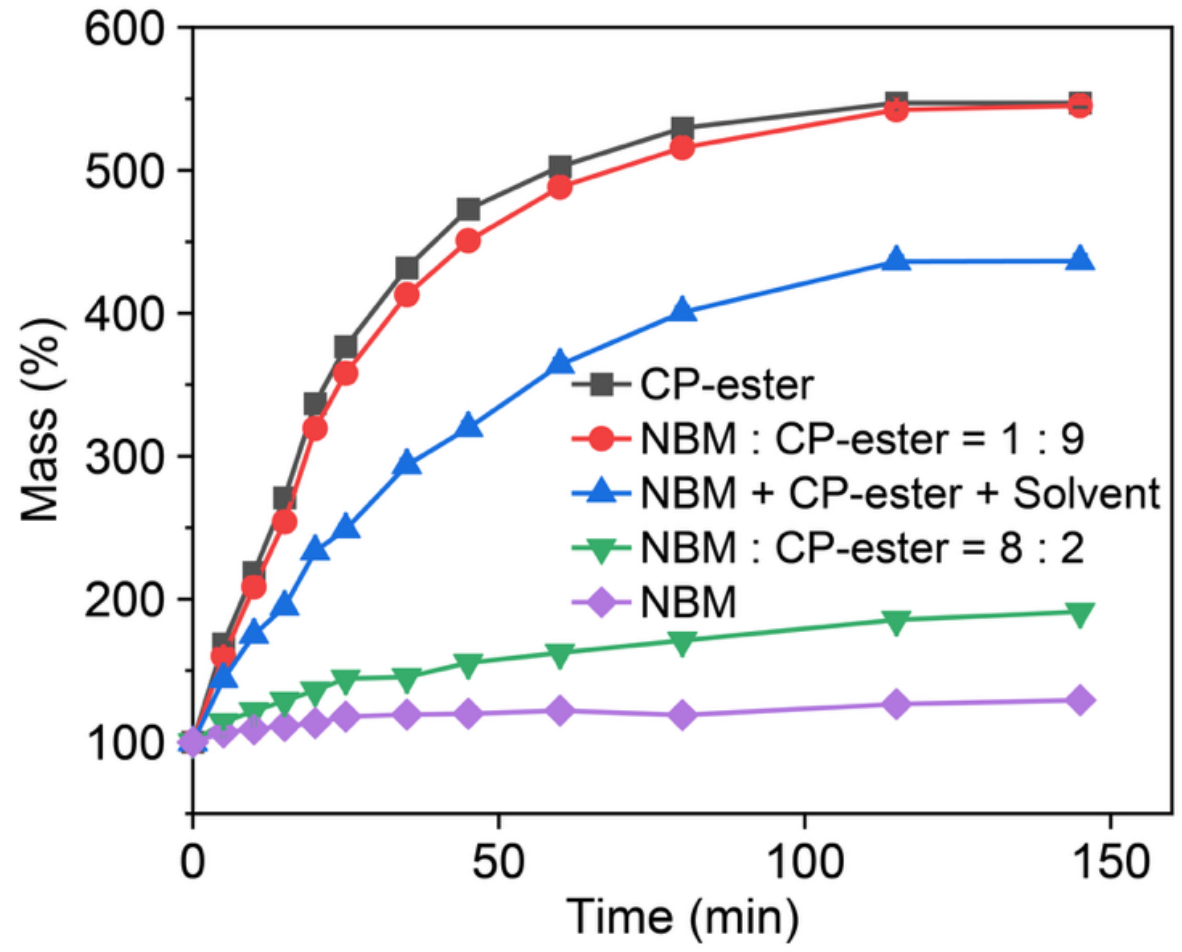

Fig. S3. Mass change along with pure swelling of a 10% NBM "living" polymer (made of 10 vol.% NBM, 88 vol.% CP-ester, 2 vol.% crosslinker, 0.5 mm thickness × 3 mm width × 3 mm length) in 100% CP-ester (noted as "CP-ester"), "NBM : CP-ester = 1:9" solution (10 vol.% NBM, 88 vol.% CP-ester, 2 vol.% crosslinker), "NBM + CP-ester + solvent" solution (40 vol.% NBM, 9 vol.% CP-ester, 1 vol.% crosslinker, 30 vol.% THF, and 20 vol.% $CHCl_3$), "NBM : CP-ester = 8:2" solution (80 vol.% NBM, 18 vol.% CP-ester, 2 vol.% crosslinker) and "NBM" (100% NBM). None of these solutions contains catalyst.

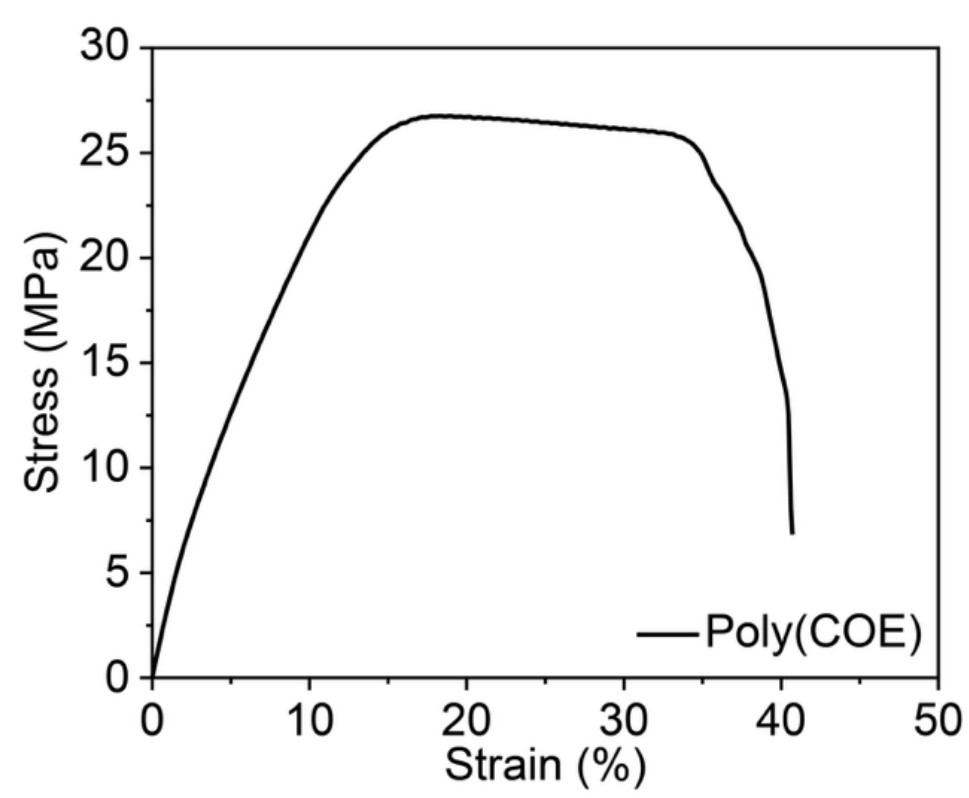

Fig. S4. Tensile property of poly(COE) (made of 98 vol.% COE, 1 vol.% CP-ester, 1 vol.% crosslinker).

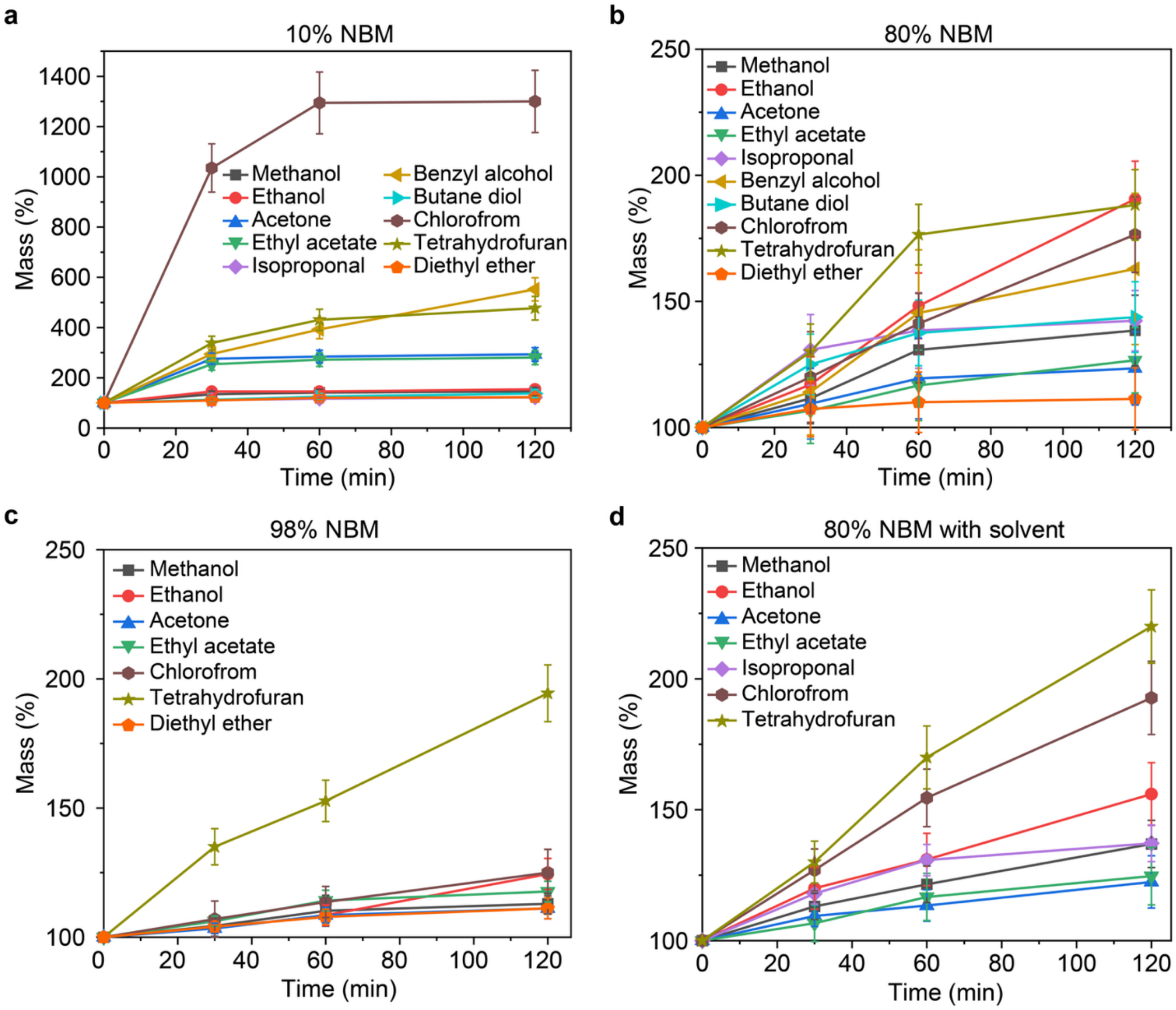

Fig. S5. The mass change over time of swelling in solvents for (**a**) 10% NBM polymer (made of 10 vol.% NBM, 88 vol.% CP-ester, 2 vol.% crosslinker), (**b**) 80% NBM polymer (made of 80 vol.% NBM, 18 vol.% CP-ester, 2 vol.% crosslinker), (**c**) 98% NBM polymer (made of 98 vol.% NBM, 2 vol.% crosslinker), and (d) 10% NBM made with solvent (made of 80 vol.% of solution A (80 vol.% NBM, 18 vol.% CP-ester, 2 vol.% crosslinker) and 20 vol.% solution B (THF : $CHCl_3$ = 3 : 2)). The samples used for swelling are in size of 0.5 mm thickness × 4 mm width × 4 mm length.

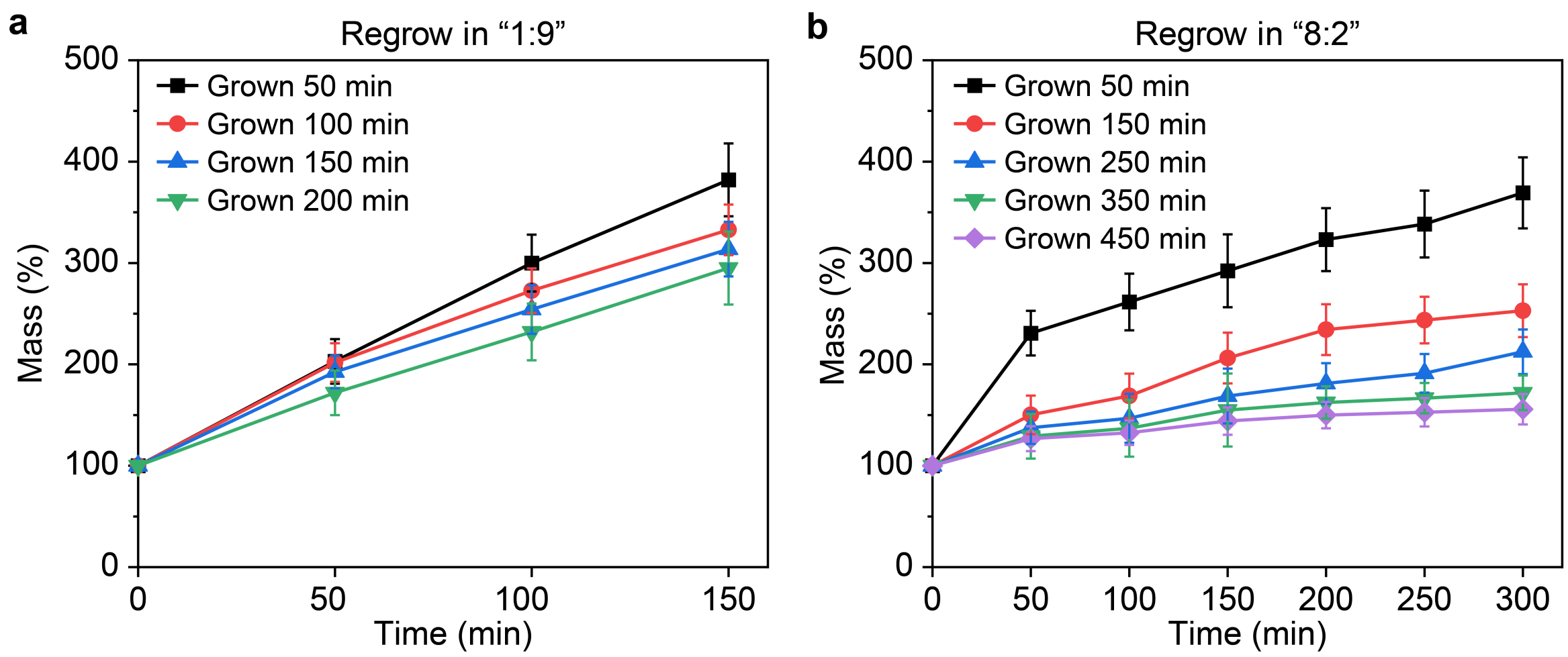

Fig. S6. **a** The mass change over regrowth time in “1:9” solution (10 vol.% NBM, 88 vol.% CP-ester, 2 vol.% crosslinker, 8mg/mL G2) for samples previously grown in “1:9” solution for a certain time duration (noted as “grown x time”). **b** The mass change over regrowth time in “8:2” solution (40 vol.% NBM, 9 vol.% CP-ester, 1 vol.% crosslinker, 30 vol.% THF, 20 vol.% $CHCl_3$, 8 mg/mL G2) for samples previously grown in “8:2” solution for varying time.

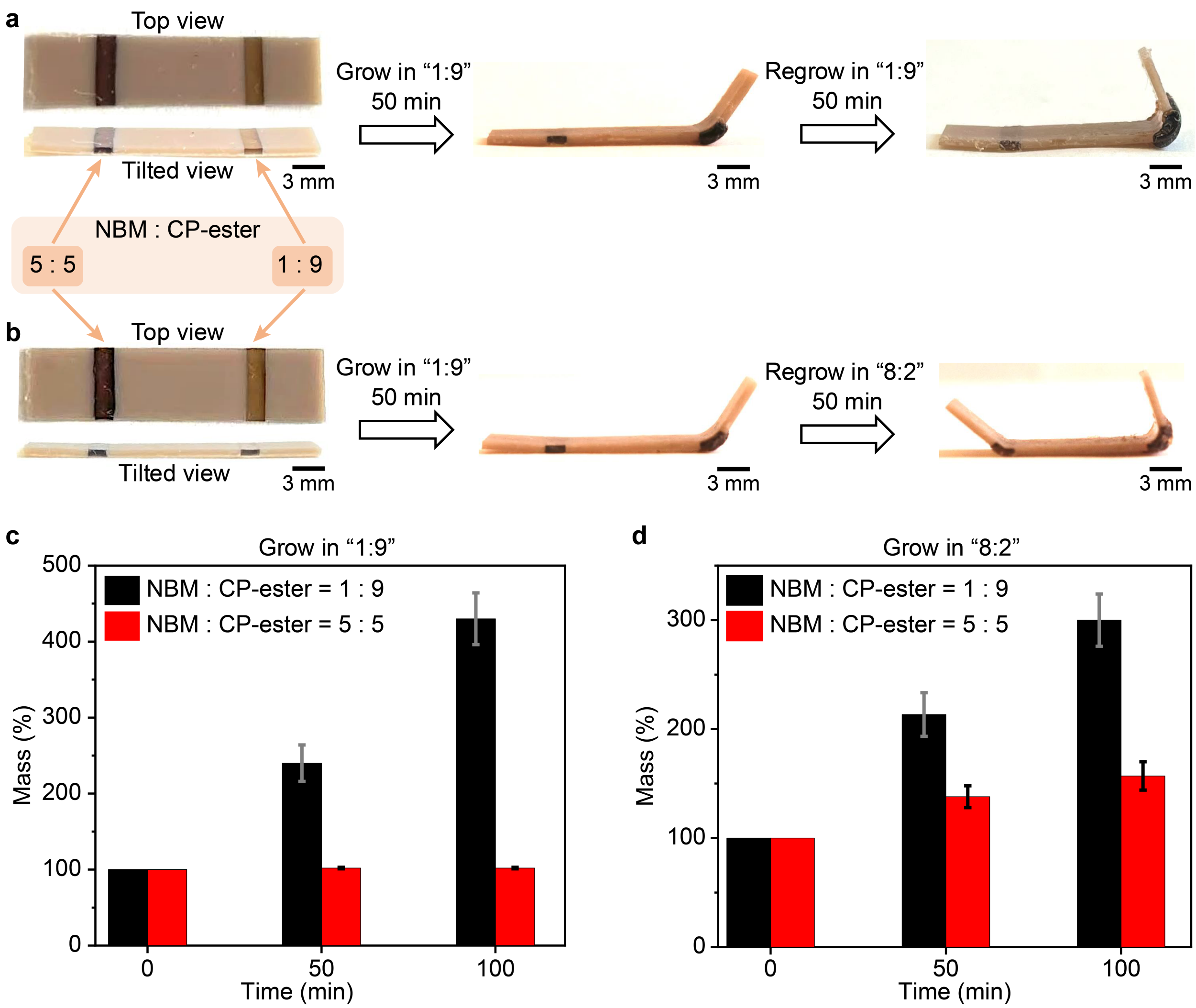

Fig. S7. a Reconfiguration of a beam structure containing two “living” polymer compositions in the two creases upon growth and regrowth in “nutrient” solution of “1:9”. **b** Reconfiguration of a beam structure containing two “living” polymer compositions in the two creases upon growth in a “nutrient” solution of “1:9” and regrowth in “8:2”. **c** The mass change of films (0.5 mm thickness × 4 mm × 4 mm) of “living” polymers made of NBM:CP-ester = 1:9 and 5:5 upon growing in a “nutrient” solution of “1:9”. **d** The mass change of films (0.5 mm thickness × 4 mm × 4 mm) of “living” polymers made of NBM:CP-ester = 1:9 and 5:5 upon growing in a “nutrient” solution of “8:2”. Scale bars in Fig. S7 all represent 3 mm.

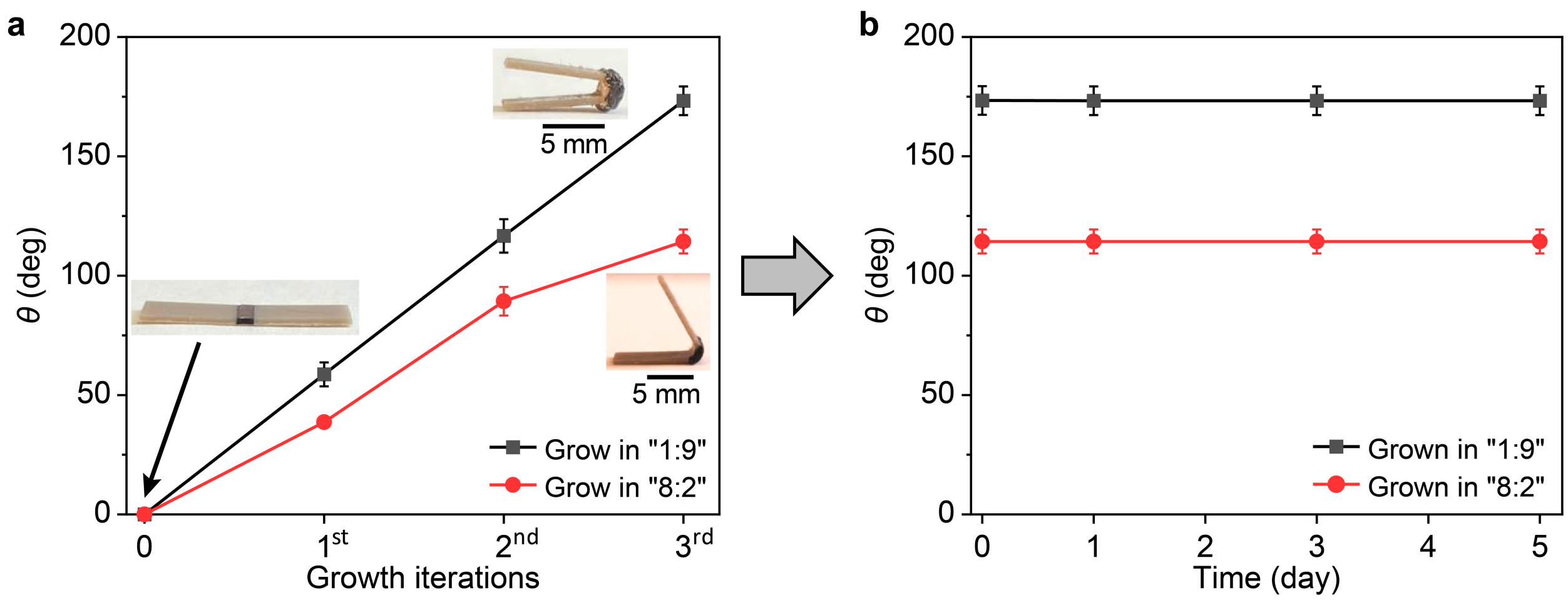

Fig. S8. **a** The change of folding angles (Θ) of the beam units over 1st growth and stop, followed by two additional times of regrowth and stop (denoted as "Growth iterations") in "nutrient" solutions of "1:9" and "2:8", respectively. The beam unit contains a "living" polymer (10% NBM, 88% CP-ester, 2% crosslinker, 1.8 mm width × 550 μm height × 6 mm length) at the crease, surrounded by passive poly(COE), with a total beam thickness of 650 μm. **b** The folding angle as a function of storage time on the bench at room temperature for the two samples after the 3rd iteration of growth in **a**. The error bar represents the standard deviation of measuring 4 independent samples.

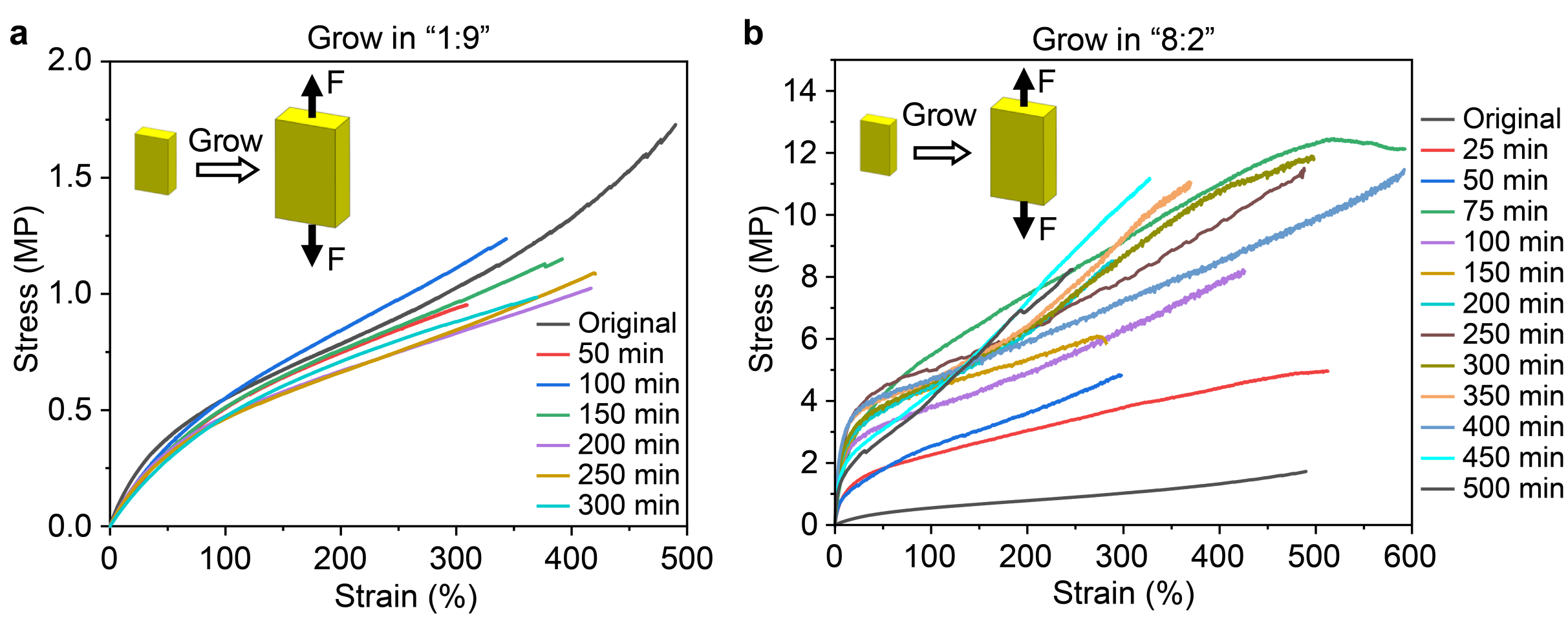

Fig. S9. a Mechanical property changes for 10% NBM "living" polymer (made of 10 vol.% NBM, 88 vol.% CP-ester, 2 vol.% crosslinker, noted as "Original") after growing in "1:9" (10 vol.% NBM, 88 vol.% CP-ester, 2 vol.% crosslinker, 8mg/mL G2) for a certation time duration. **b** Mechanical property changes for 10% NBM "living" polymer over growth in "8:2" (40 vol.% NBM, 9 vol.% CP-ester, 1 vol.% crosslinker, 30 vol.% THF, 20 vol.% $CHCl_3$, 8 mg/mL G2) for a certation time duration.

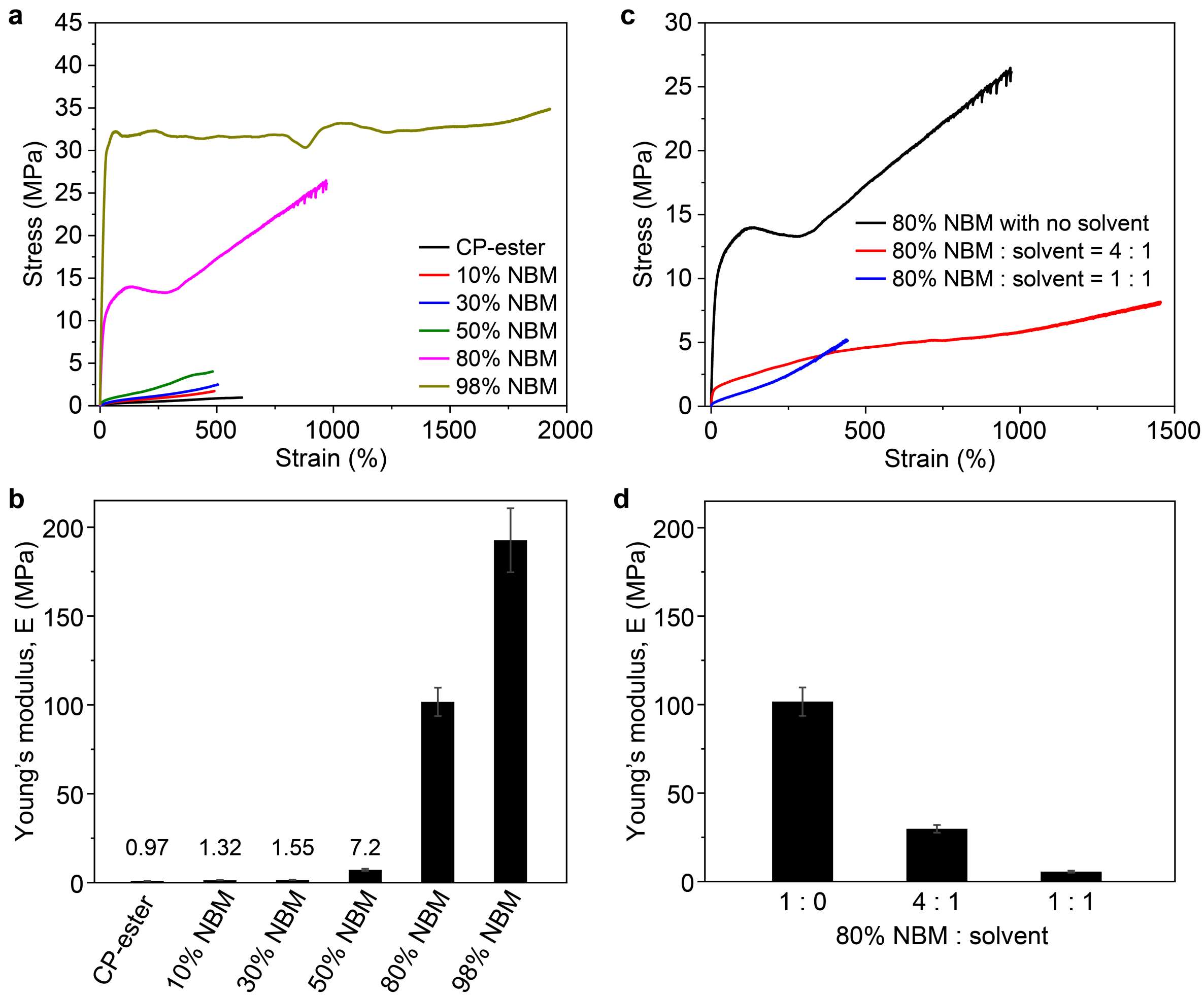

Fig. S10. a Tensile mechanical properties of poly(CP-ester) with 2 vol.% crosslinker (noted as "CP-ester") and copolymers of CP-ester and NBM with varying amounts of NBM. Here, "x% NBM" indicates a sample made of x vol.% NBM, (100-x-2) vol.% CP-ester, 2 vol.% crosslinker. The crosslinker concentrations in all samples are 2 vol.%. **b** The Young's modulus of the samples with varying NBM percentage, extracted from the tensile property in Fig. S10a. **c** The tensile mechanical properties of the samples prepared with and without solvent (60 vol.% THF, 40 vol.% $CHCl_3$). **d** The Young's modulus of the samples in Fig. S10c.

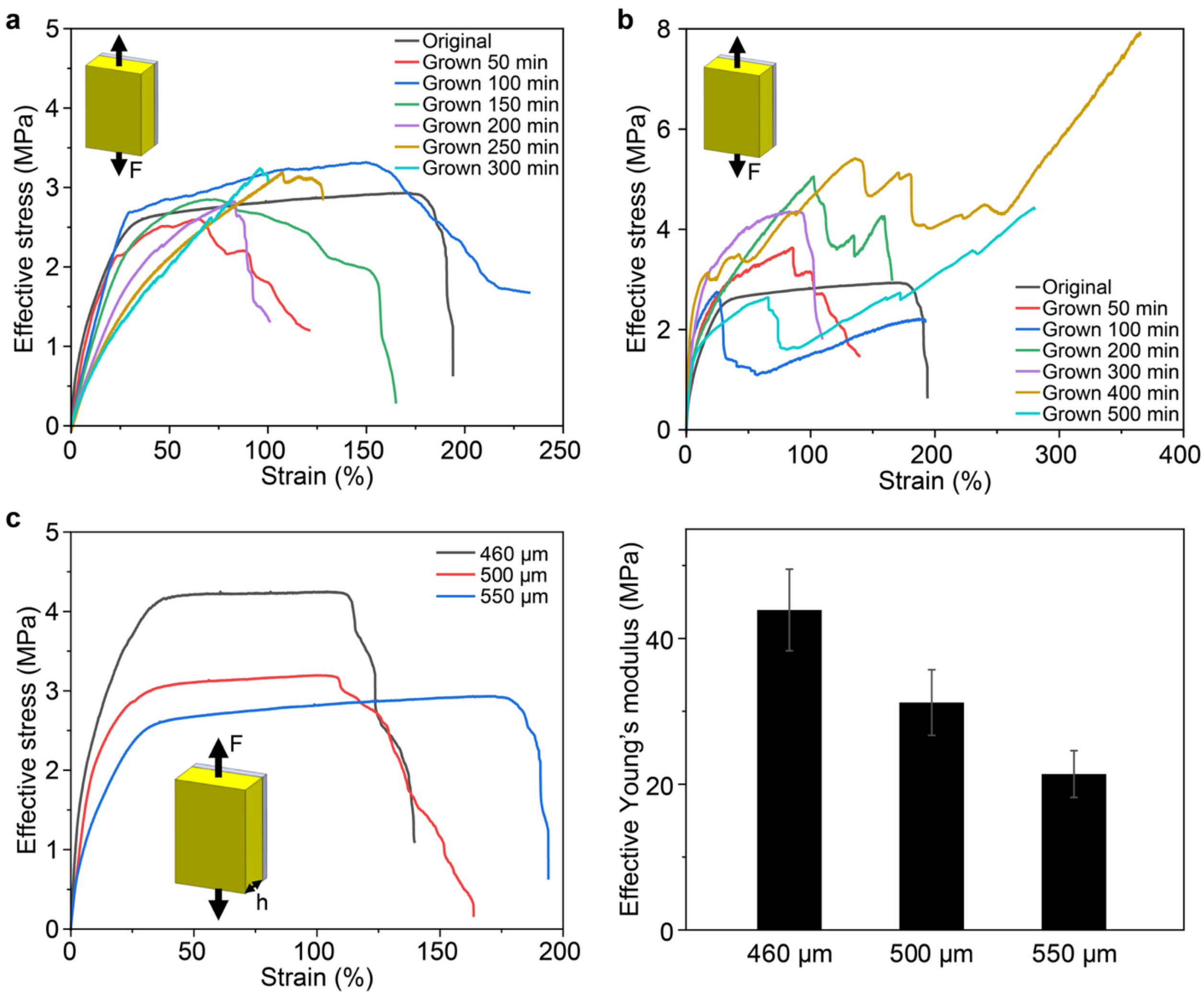

Fig. S11. a Tensile properties of the crease mimicking two-layer structures composed of 10% NBM "living" polymer (yellow part, made of 10 vol.% NBM, 88 vol.% CP-ester, 2% crosslinker) and passive poly(COE) (grey color, always 100 μm thick). "Original" indicates the "living" polymer of 550 μm thickness. "Grow 50 min" indicates the "living" polymer (originally 550 μm thick) that has grown in "1:9" solution for 50 min before bonding with the poly(COE) layer, and the same naming principle applies to other growth times. **b** Similar naming rule as that in **a**, with the same material as "Original". The difference being that the growth is in an "8:2" solution. **c** Left: Tensile properties of the two-layer crease-mimicking structures with varying thickness of 10% NBM "living" polymer (noted as "xx μm" in the legend, yellow part in the schematic) and the poly(COE) layer (yellow part in the schematic), while keeping the total thickness of 650 μm. The Effective stress is calculated as force divided by the initial sample cross-sectional area. Right: The effective Young's modulus (effective stress divided by strain) of the samples.

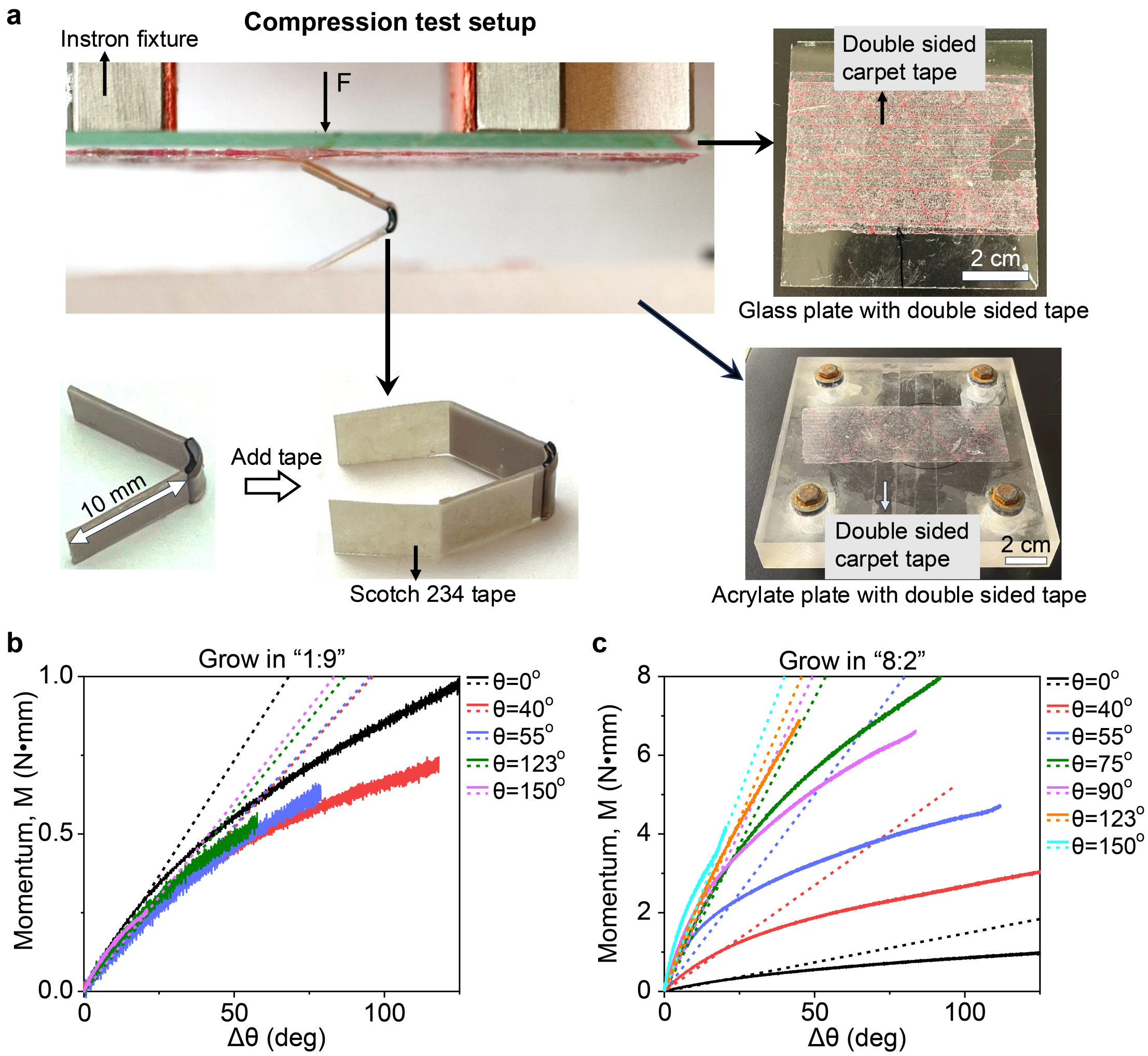

Fig. S12. a The compression test setup. **b** The momentum as a function of *Δω* for beam units grown in "1:9" solution (10 vol.% NBM, 88 vol.% CP-ester, 2 vol.% crosslinker, 8mg/mL G2) to a certain folding angle ϴ. The data was measured under the compression test. The linear regression is based on the first 20 degrees of Δ*ω*. **c** The momentum as a function of *Δω* for beam units grown in "8:2" solution (40 vol.% NBM, 9 vol.% CP-ester, 1 vol.% crosslinker, 30 vol.% THF, 20 vol.% $CHCl_3$, 8 mg/mL G2) to a certain folding angle ϴ, and the data fitting of the first 20 degrees. Solid lines: experimental data. Dash lines: linear regression.

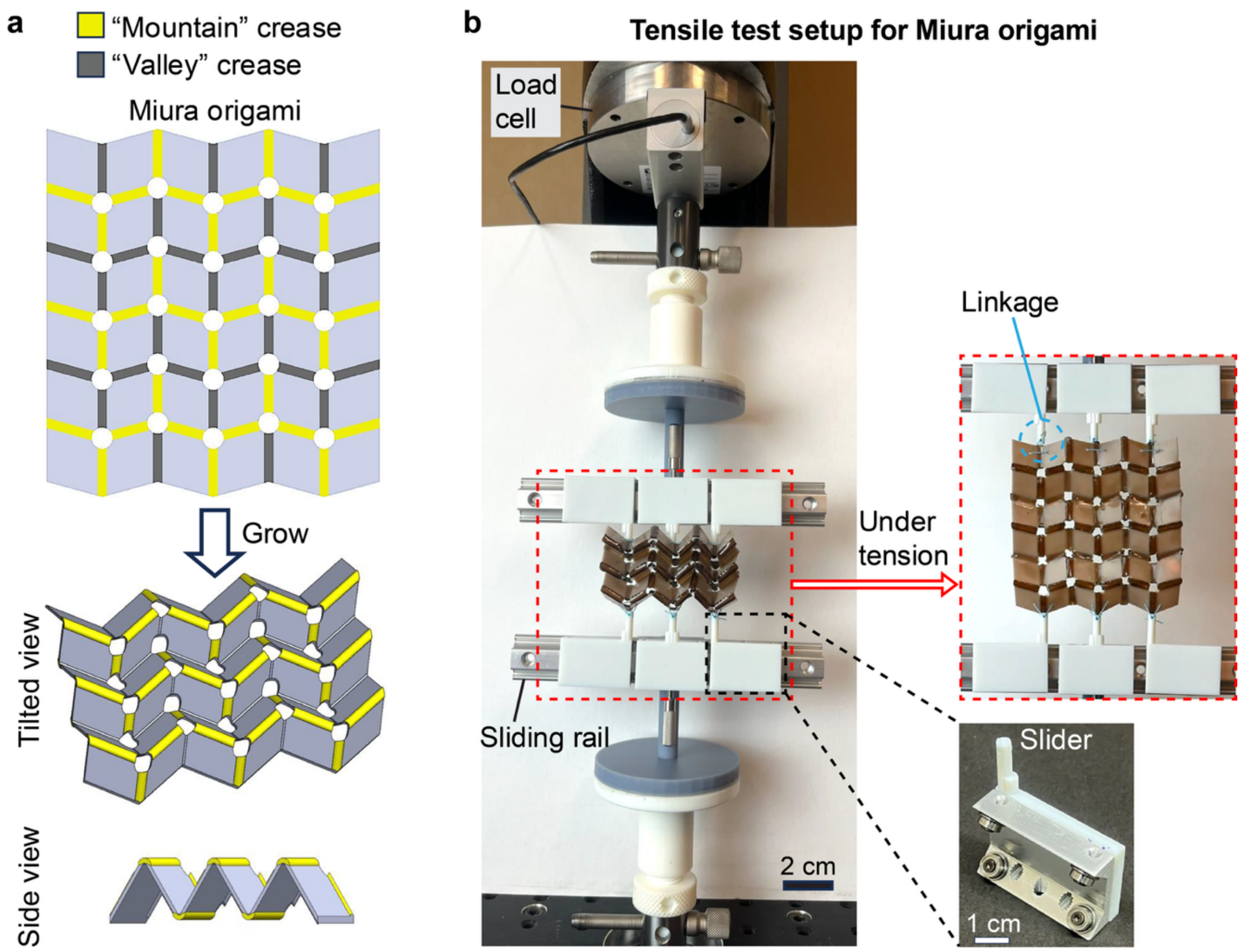

Fig. S13. a Schematics of the Miura origami design and growth induced self-folding of the Miura origami. **b** The setup for measuring the tensile properties of the Miura origami.

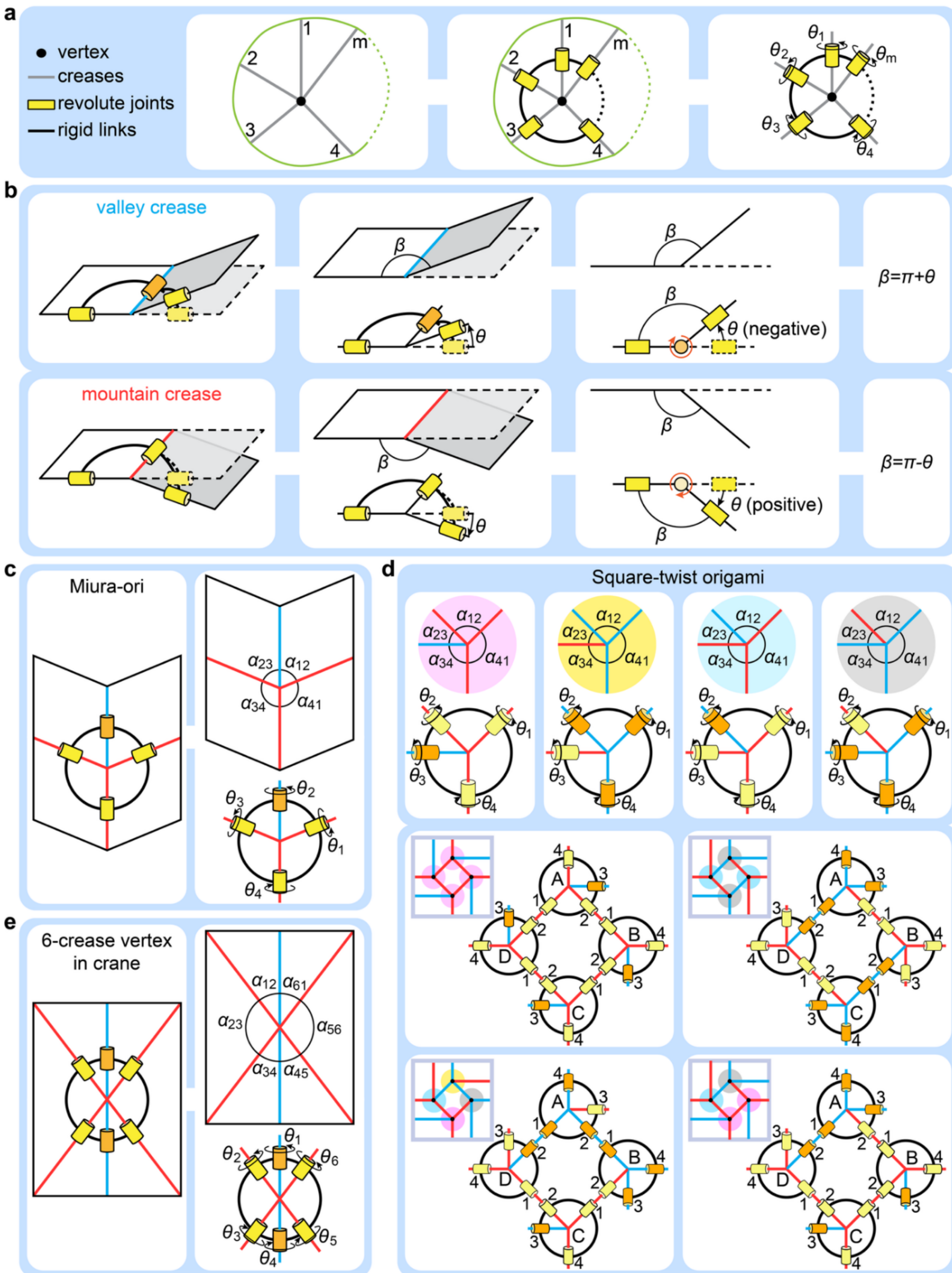

Fig. S14. Kinematic analysis of origami structures. **a** An *m*-crease vertex and the corresponding spherical *mR* linkage. **b** Definition of valley and mountain creases and the corresponding joints and links. **c** Miura-ori pattern modelled by a spherical 4*R* linkage. **d** Square-twist origami patterns modelled by spherical 4*R* linkages. **e** The 6-crease vertex in the crane modelled by a spherical 6*R* linkage.

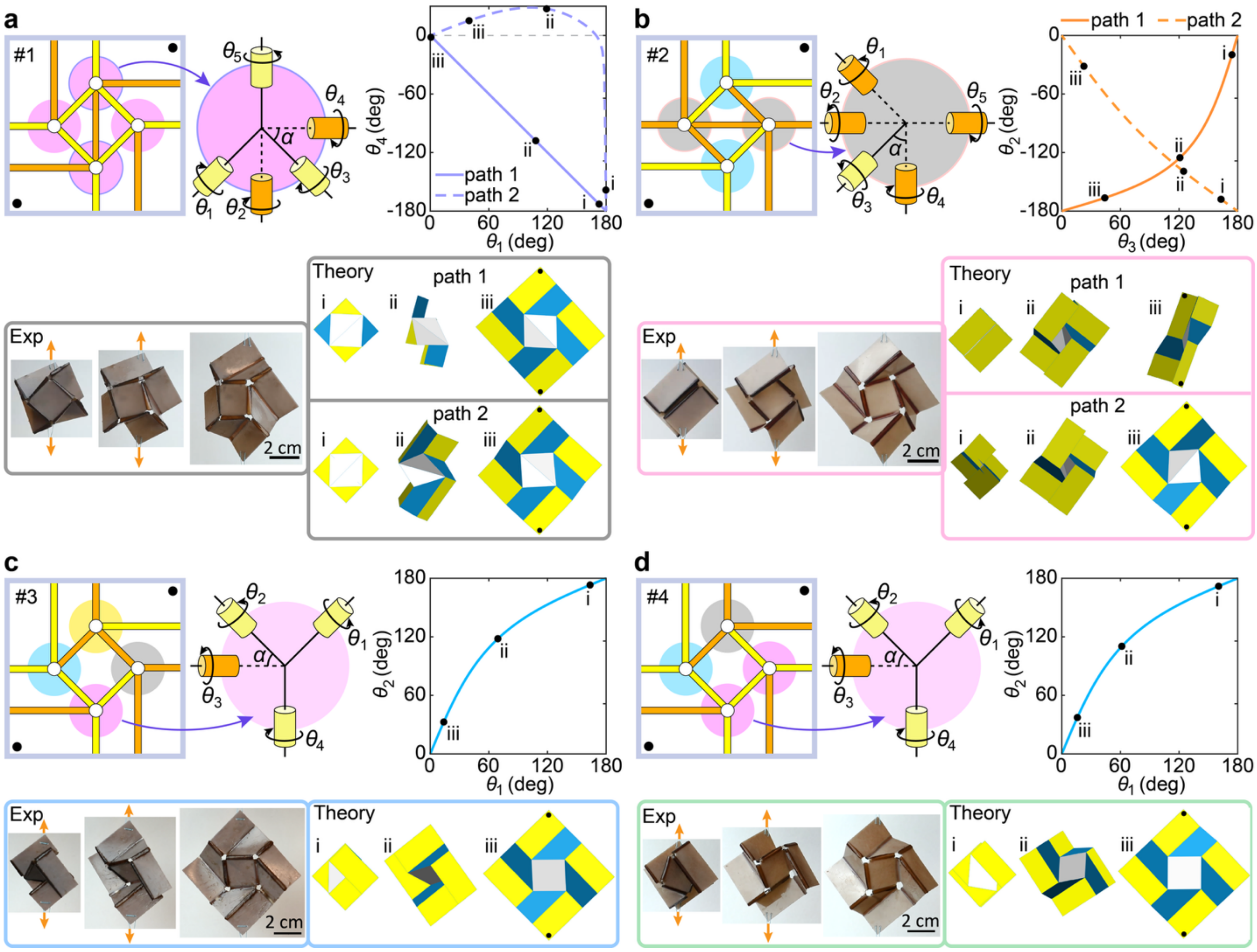

Fig. S15. Unfolding process of square-twist structures. **a** Relationship between rotational angles θ_4 and θ_1 for the modified non-rigid square-twist cell #1 with an added crease in the central square, together with the experimental snapshots and the theoretical unfolding prediction. **b** Relationship between rotational angles θ_2 and θ_3 for the modified non-rigid square-twist cell #2 with an added crease in the central square, together with the experimental snapshots and the theoretical unfolding prediction. **c** Relationship between rotational angles θ_2 and θ_1 for the rigid square-twist cell #3, together with the experimental snapshots and the theoretical unfolding prediction. **d** Relationship between rotational angles θ_2 and θ_1 for rigid square-twist cell #4, together with the experimental snapshots and the theoretical unfolding prediction.

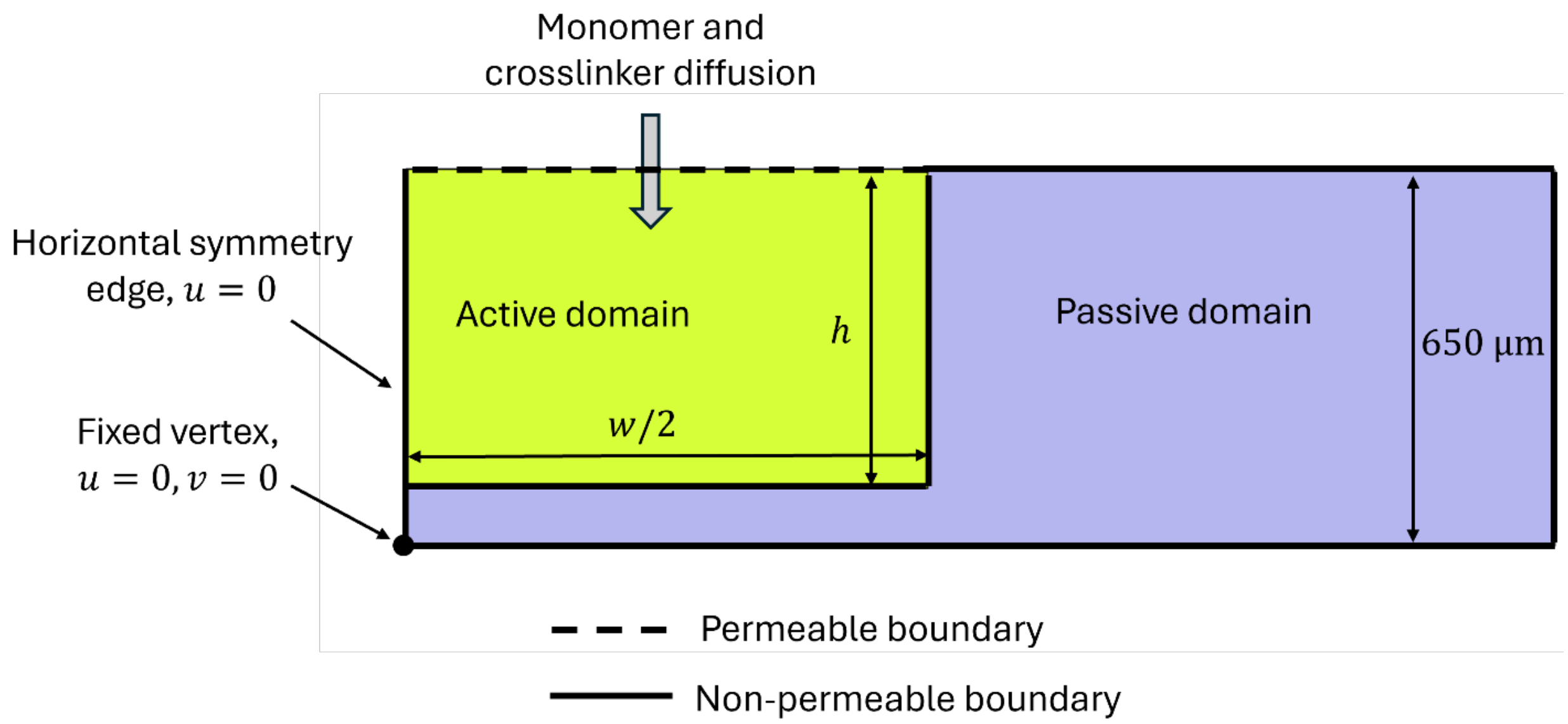

Fig. S16. Geometry and boundary condition schematic. The active domain is shown in yellow color, while the passive domain is in blue color. Monomer and crosslinker can diffuse in through the top edge. Only the active domain top edge is permeable, rest all boundary or interfaces edges are non-permeable. The left edge has symmetric boundary conditions, that is no horizontal displacement and not species transport. While the bottom left vertex is fixed. The total sample height is kept fixed at $h_T = 650\ \mu m$. Parametric studies are performed on the active domain height and width.

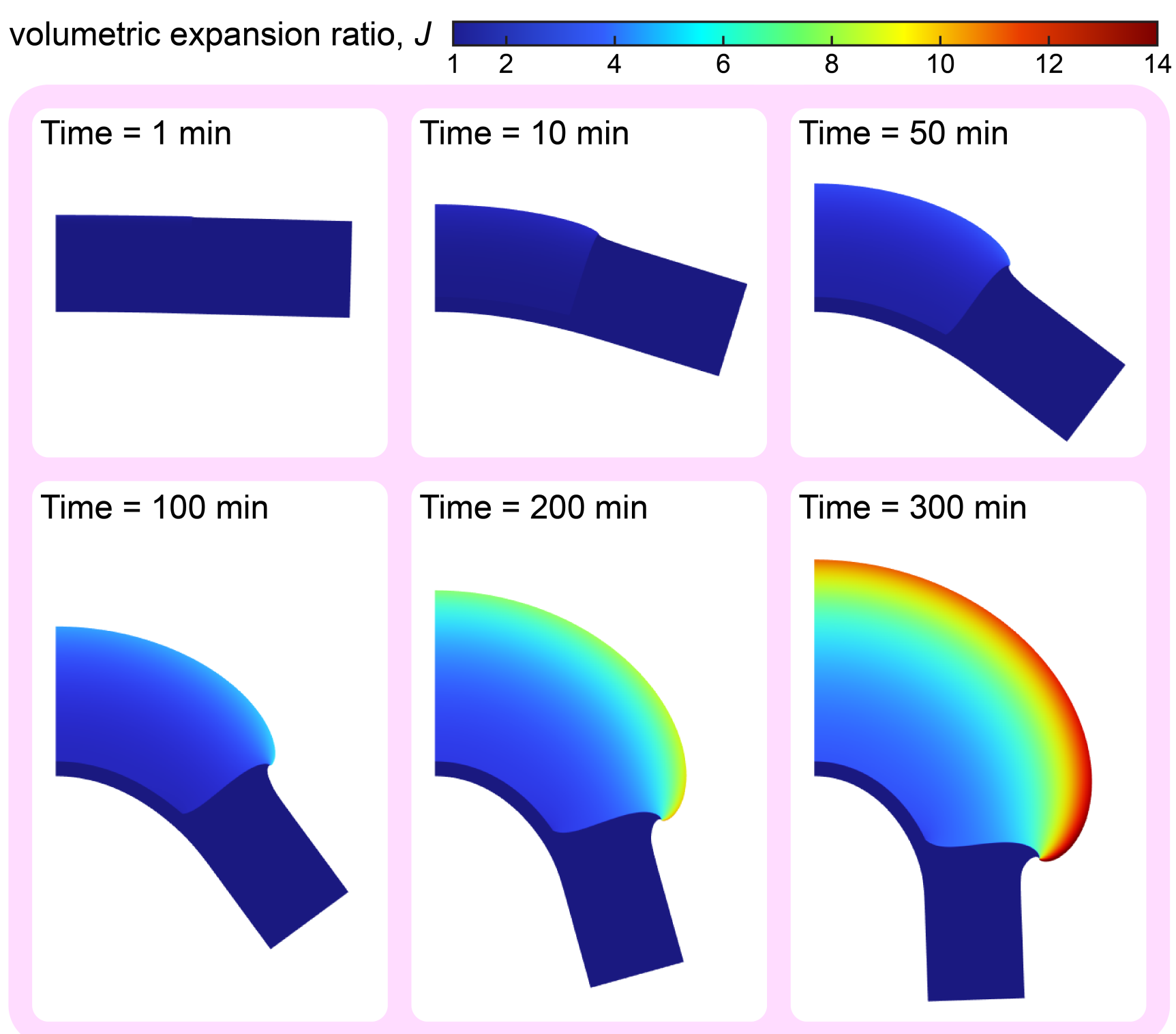

Fig. S17. Panel folding simulation snapshots in the growth process. The contour plots represent the volume expansion ratio. The folding angle changes from approximately 0° at 1 minute of growth to almost 180° at 300 minutes of growth.

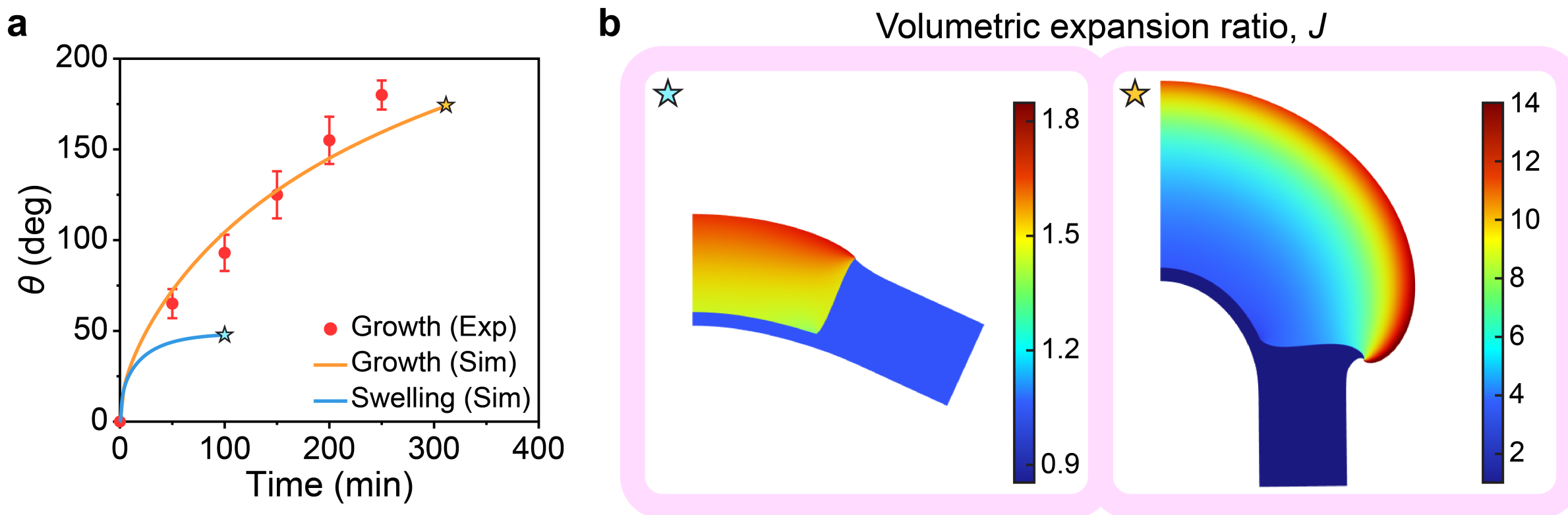

Fig. S18. Growth vs swelling simulation comparison. a) The experiments and simulation showing the folding angle change for scenarios of swelling and growth; b) The volumetric expansion ratios and the contour plots for scenarios of swelling (left) and growth (right). A volumetric change of more than 1 order of magnitude is seen in growth simulations. The sample dimensions are: $w = 1.8\ mm, h = 550\ \mu m$.

References

[1] J. Denavit, R. S. Hartenberg, "A kinematic notation for lower-pair mechanisms based on matrices", *Journal of Applied Mechanics* **1955**, 22, 215

[2] Y. Chen, H. Feng, J. Ma, R. Peng, Z. You, "Symmetric waterbomb origami", *Proceedings of the Royal Society A: Mathematical, Physical and Engineering Sciences* **2016**, 472, 20150846

[3] H. Feng, R. Peng, S. Zang, J. Ma, Y. Chen, "Rigid foldability and mountain-valley crease assignments of square-twist origami pattern", *Mechanism and Machine Theory* **2020**, 152, 103947

[4] J. Ma, S. Zang, H. Feng, Y. Chen, Z. You, "Theoretical characterization of a non-rigid-foldable square-twist origami for property programmability", *International Journal of Mechanical Sciences* **2021**, 189, 105981
